# Tuning the Drug Efflux Activity of an ABC Transporter *in* *vivo* by *in vitro* Selected DARPin Binders

**DOI:** 10.1371/journal.pone.0037845

**Published:** 2012-06-04

**Authors:** Markus A. Seeger, Anshumali Mittal, Saroj Velamakanni, Michael Hohl, Stefan Schauer, Ihsene Salaa, Markus G. Grütter, Hendrik W. van Veen

**Affiliations:** 1 Department of Pharmacology, University of Cambridge, Cambridge, United Kingdom; 2 Department of Biochemistry, University of Zurich, Zurich, Switzerland; 3 Functional Genomics Center Zurich, University of Zurich, Zurich, Switzerland; Technion-Israel Institute of Technology, Israel

## Abstract

ABC transporters use the energy from binding and hydrolysis of ATP to import or extrude substrates across the membrane. Using ribosome display, we raised designed ankyrin repeat proteins (DARPins) against detergent solubilized LmrCD, a heterodimeric multidrug ABC exporter from *Lactococcus lactis*. Several target-specific DARPin binders were identified that bind to at least three distinct, partially overlapping epitopes on LmrD in detergent solution as well as in native membranes. Remarkably, functional screening of the LmrCD-specific DARPin pools in *L. lactis* revealed three homologous DARPins which, when generated in LmrCD-expressing cells, strongly activated LmrCD-mediated drug transport. As LmrCD expression in the cell membrane was unaltered upon the co-expression of activator DARPins, the activation is suggested to occur at the level of LmrCD activity. Consistent with this, purified activator DARPins were found to stimulate the ATPase activity of LmrCD *in* *vitro* when reconstituted in proteoliposomes. This study suggests that membrane transporters are tunable *in vivo* by *in vitro* selected binding proteins. Our approach could be of biopharmaceutical importance and might facilitate studies on molecular mechanisms of ABC transporters.

## Introduction

In the past decade, unprecedented progress has been made in the elucidation of ten complete ABC transporter structures solved by X-ray crystallography, which guide current functional studies on these transport proteins [1]–[5]. However, the mechanisms of transport of both, ABC importers and exporters are still controversial [6]. One reason for the uncertainties is due to the fact that crystal structures represent snapshots of the proteins in specific conformations. In order to describe the transport cycle in detail, several structures of the same transporter captured in different conformational states need to be solved. This often requires the trapping of the transport protein in a specific conformational state which, for crystallized primary-active transporters, was achieved by using non-hydrolyzable nucleotide analogs such as AMP-PNP [7], [8] or various nucleotide trapping agents such as vanadate, aluminium fluoride and beryllium fluoride [9], [10], or by generating mutant proteins that are unable to hydrolyze ATP [11]. However, as these different techniques interrupt the catalytic cycle of ATP hydrolysis at similar stages, the repertoire of conformations that can be stabilized is limited.

To overcome this limitation, we used designed ankyrin repeat proteins (DARPins) which represent a novel binding scaffold [12]. DARPins typically consist of two or three internal ankyrin repeat units encoding the randomized surface flanked by an N-terminal and a C-terminal capping repeat [13], [14]. DARPins are devoid of disulfide bonds, easy to produce in *E. coli* and extraordinarily robust [15]. High-affinity binders have been raised against a growing number protein targets [16], [17]. Amongst these is an AcrB specific DARPin that was co-crystallized with AcrB to obtain the highest resolution structure at 2.5 Å of this membrane protein to date [18], [19].

Traditionally, monoclonal antibodies (mAbs) specific for integral membrane proteins have been generated using the hybridoma technology [20]. This procedure relies on the natural generation of binders against the targeted protein in mice [21]–[26]. However, the process of binder selection after the injection of the protein sample into the animal is beyond experimental control. *In* *vitro* selections using either phage display or ribosome display in contrast allow binder selection under defined conditions [27], [28]. Nevertheless, the small number of less than ten published studies on the complete *in vitro* selection of binders (Fab fragments and DARPins) against detergent-purified membrane proteins embodies the difficulties in using membrane proteins for this purpose [18], [29]–[36].

ABC transporters play a pivotal role in the active transport of molecules in organisms of all kingdoms of life. The mammalian multidrug transporter ABCB1 (also termed P-glycoprotein or MDR1) has probably attracted most attention of all ABC transporters, since it can play an important role in the extrusion of noxious substances out of the cell, and has been linked to drug resistance in tumor cells [37], [38]. Prokaryotic homologues of ABCB1 such as LmrA from *Lactococcus lactis* and MsbA from *Escherichia coli*, and analogues such as LmrCD from *L. lactis* were studied in detail and are involved in the transport of drugs, lipids and small ions [39]–[45]. ABC transporters use the energy of ATP binding and hydrolysis catalyzed by the nucleotide binding domains (NBDs) to translocate substrates through the membrane domain (MDs). For this purpose, the NBDs need to dimerize in a sandwich-like fashion forming two composite catalytic sites [46]. The amino acids involved in ATP binding and hydrolysis are encoded by a number of highly conserved sequence motifs including Walker A, Walker B, ABC Signature and H-loop (for review, see [2]).

In LmrCD, one of the two composite catalytic sites at the NBDs deviates from the consensus sequence and is postulated to mediate ATP binding, but not ATP hydrolysis [47]. The deviation from the canonical sequence concerns the catalytically important Walker B glutamate and H-loop histidine that are changed to aspartate and glutamine, respectively. The same substitutions are found in the non-canonical sites of the antigen peptide transporter TAP1/2 and the yeast multidrug transporter Pdr5 [48], [49]. Here, we demonstrate the successful *in vitro* selection of binders against detergent-solubilized LmrCD using ribosome display. Moreover, we use the lactococcal cells for a novel *in vivo* functional screen applicable for multidrug transporters, and we characterize the functional consequences of DARPin binding to LmrCD.

## Results

### Selection of DARPins Against Detergent Solubilized LmrCD

**Figure 1:**
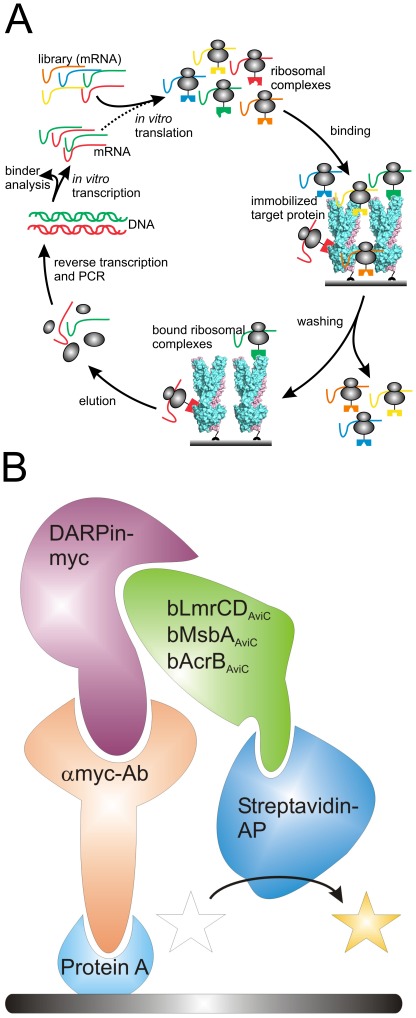
Ribosome display and ELISA set-up. (**A**) Sketch delineating one DARPin selection round using ribosome display (adopted from [31]). The DARPin library in form of mRNA is *in vitro* translated and stable ribosomal complexes linking the phenotype (folded DARPins) with the genotype (translated mRNA) are generated. The ribosomal complexes are allowed to bind to immobilized bLmrCD_AviC_. After a washing step of variable length (depending on selection stringency), bound ribosomal complexes are destabilized and mRNA encoding for potential target-specific DARPins is liberated. The eluted mRNA is amplified by reverse transcription and PCR to double stranded DNA, which is *in vitro* transcribed into mRNA for another round of selection or used for binder analysis. (**B**) Schematic drawing of the ELISA set up. Protein A is coated onto the ELISA well and is decorated with an anti-myc antibody that immobilizes the DARPins via the C-terminal Myc5-tag. Upon binding of purified, biotinylated target protein (e.g. LmrCD, AcrB or MsbA in our study) to DARPin, the target protein is detected using a streptavidin-alkaline phosphatase the activity of which was detected colourimetrically at OD_405_ using p-nitrophenyl phosphate as a substrate.

We cloned the *lmrCD* genes with a His10-tag N-terminally to LmrC, and were able to purify functionally active LmrCD to homogeneity from lactococcal membrane vesicles. The proteins could be isolated as heterodimeric species from size exclusion chromatography (SEC) columns (Figure S1A and B). Interestingly, the heterodimeric complex of LmrCD was stable when the purified protein was analyzed by nano-electrospray mass spectrometry [50]. In order to immobilize LmrCD during the DARPin selection procedure, an Avi-tag was introduced C-terminally to LmrD, which allowed for site-specific enzymatic biotinylation of a lysine residue comprised within the Avi-tag sequence (biotinylated LmrCD is denoted bLmrCD_AviC_) [51]. The DARPin selection was performed using the ribosome display method with DARPins including three internal randomized repeats (N3C DARPins) (Figure 1A) [12], [18], [28]. A total of 4 sequential selection rounds were performed in which catalytically active bLmrCD_AviC_ and orthovanadate-trapped bLmrCD_AviC_ were used as two independent protein formulations. In the presence of 1 mM ATP, LmrCD could be trapped by orthovanadate with a concentration giving half-maximal inhibition of ATP hydrolysis (IC_50_) of 120 µM which is in agreement with a recent study on the heterodimeric ABC transporter BmrCD [52] (data not shown). The orthovanadate concentration (1 mM) used during the DARPin selections comfortably exceeded this IC_50_. It should be noted that around 0.6 mM of ATP originating from the *in vitro* translation buffer and around 40 mM magnesium acetate were present during the incubation of the DARPins with the target protein. This means that in case of the non-trapped bLmrCD_AviC_ formulation, the DARPins were selected against transporters slowly hydrolyzing ATP and presumably adopting various conformational states.

### Identification of LmrCD-specific DARPin Binders by ELISA

**Figure 2:**
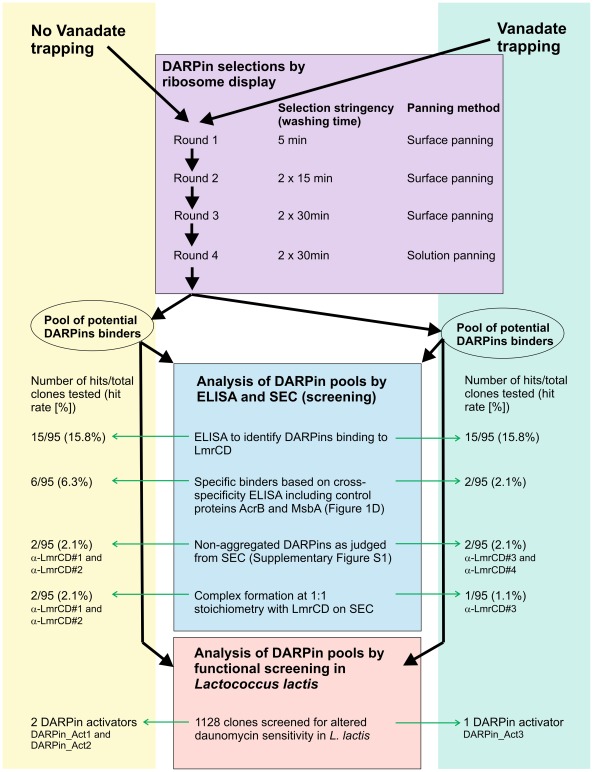
Workflow of DARPin selection and screening. DARPins were selected by ribosome display against LmrCD with and without vanadate trapping (purple rectangle). After four sequential selection rounds of increasing stringency, the pools of potential binders were analyzed either by ELISA and SEC (blue rectangle) or in a functional screen in *L. lactis* (red rectangle).

**Figure 3:**
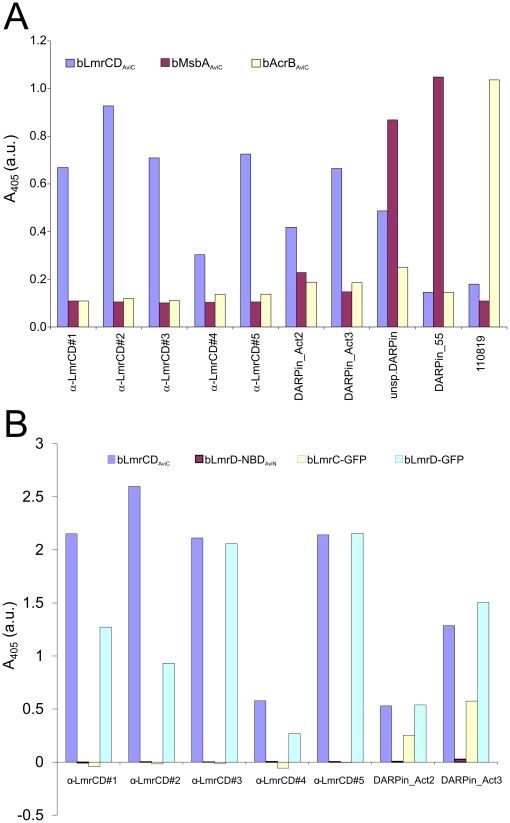
Identification and characterization of DARPin binders by ELISA (**A**) Specificity ELISA using bLmrCD_AviC_, bMsbA_AviC_ and bAcrB_AviC_ as target proteins. Seven DARPins (α-LmrCD#1-5, DARPin_Act2 and DARPin_Act3) were found to be highly specific for bLmrCD_AviC_. Many initial DARPin binder-hits promiscuously bound to bLmrCD_AviC_, bMsbA_AviC_ and bAcrB_AviC_ as exemplified with the “unsp. DARPin” and were therefore not useful for further analysis. DARPins specific for bMsbA_AviC_ (DARPin_55) and bAcrB_AviC_ (110819) were used as a positive control. (**B**) ELISA analyzing binding of the LmrCD-specific DARPins shown in (A) to LmrC (bLmrC-GFP), LmrD (bLmrD-GFP) and the nucleotide binding domain of LmrD (bLmrD-NBD_AviN_). Binding to LmrCD (bLmrCD_AviC_) was confirmed as positive control.

We analyzed 190 clones from the DARPin pools, enriched over four selection rounds against untreated or vanadate-trapped bLmrCD_AviC_, by an established ELISA protocol (95 DARPins for each protein formulation) (Figure 1B, Figure 2) [31]. From the initial ELISA (not shown) we chose the clones giving rise to the 30 most intense ELISA signals against bLmrCD_AviC_ (15.8% of examined clones) for a second comparative ELISA (Figure 3A). Besides LmrCD, the ABC transporter MsbA and the secondary-active multidrug transporter AcrB were used in the assay (prepared as proteins biotinylated at the C-terminal Avi-tag). From the 30 ELISA-positive DARPins, 8 were exclusively binding to bLmrCD_AviC_ but not to bMsbA_AviC_ or bAcrB_AviC_ (4.2% of all examined clones), whereas the other 22 DARPins were promiscuously binding to all membrane proteins used in the specificity ELISA (Figure 3A). The quality of the control proteins bMsbA_AviC_ and bAcrB_AviC_ was confirmed by using target-specific DARPins in the ELISA assay (AcrB-specific DARPin 110819 is described [18]; the selection of the MsbA-specific DARPin_55 will be published elsewhere). The genes encoding the eight LmrCD-specific DARPins were sub-cloned, expressed without the C-terminal Myc5-tag and analyzed by size exclusion chromatography. Four of these DARPins displayed a substantial degree of aggregation (soluble aggregates) and were therefore excluded. The other four LmrCD-specific DARPins (α-LmrCD#1-4) ran as monomeric or dimeric species on SEC taking the elution profile of the monomeric control DARPin E3_5 as a reference (Table 1, Figure S1C). Three out of these four DARPins exhibited tight binding to purified LmrCD, and eluted in complex with their target from the size exclusion column. Thus, the initially chosen 190 DARPin clones could be narrowed down to 3 specific high-affinity binders, corresponding to a hit rate of 1.6%. A fifth high-affinity DARPin (α-LmrCD#5) was found in another ELISA screen identical to the one above (not shown).

**Table 1:** Biophysical properties of LmrCD-specific DARPins.

DARPin	Oligomeric state a)	Binding stoichiometry (DARPin: LmrCD) b)	*K*_D_ (nM) c)	k_a_ (×10^5^ M^−1^s^−1^) c)	k_d_ (×10^−2^ s^−1^) c)	*K*_D, eq._ (nM) d)
*Binders*						
α-LmrCD#1	Monomer	1.11∶ 1	10.0	7.40	0.738	10.7
α-LmrCD#2	Dimer/Trimer	0.96∶ 1	3.9	5.29	0.205	9.2
α-LmrCD#3	Monomer	0.73∶ 1	53.4	12.3	6.59	53.5
α-LmrCD#4	Monomer	No complex	167	2.00	3.34	173
α-LmrCD#5	Monomer	0.75∶ 1	43.0	5.10	2.19	45.2
*Activators*						
DARPin_Act1	Soluble aggregates	n.d. e)	n.d.	n.d.	n.d.	n.d
DARPin_Act2	Hexamer	n.d. e)	46.7	0.17	0.079	66.8
DARPin_Act3	Monomer	1.16∶ 1	50.5	4.36	2.20	54.9

a) Derived from elution volume of main peak on Superdex 200 10/300 GL column (Figure S1).

b) Determined by protein chip analysis (Figure 7B).

c) Values obtained by SRP analysis using a 1∶1 binding model (Figure 7C).

d) Value obtained by SPR analysis using binding equilibrium data (Figure 7D).

e) Separation of the DARPin-LmrCD complex from DARPin aggregates was not possible on SEC (Figure S1).

### Identification of Activators of LmrCD by Functional Screening in L. Lactis

**Figure 4:**
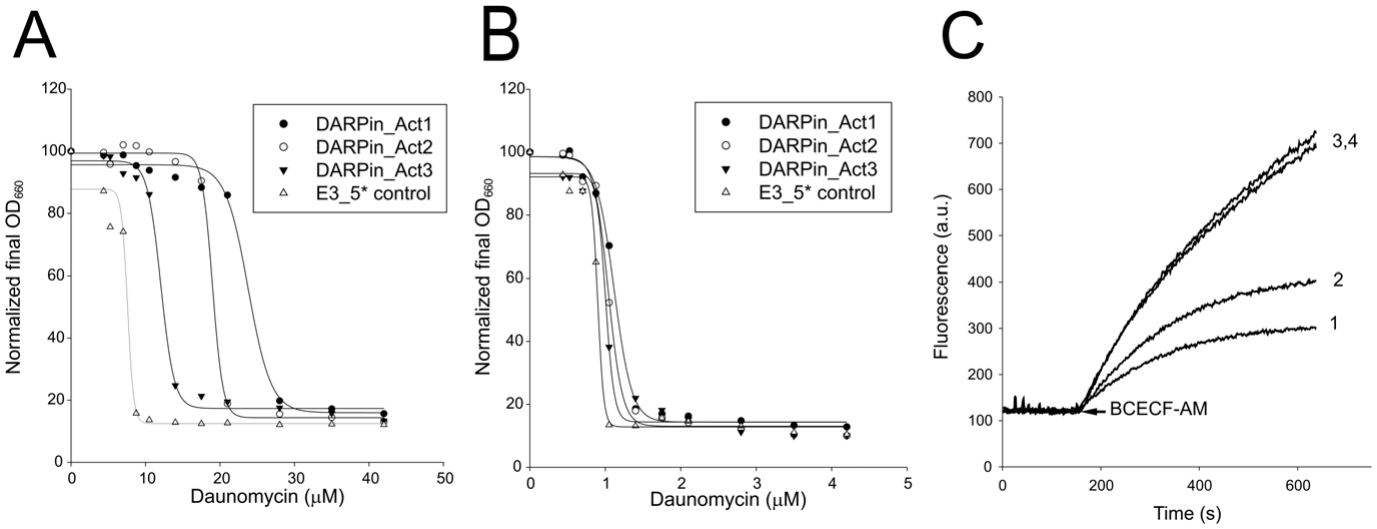
Identification of LmrCD-activating DARPins. (**A**) Overexpression of DARPin_Act1 (•), DARPin_Act2 (○), DARPin_Act3 (▾) in wildtype *L. lactis* increases the resistance towards daunomycin compared to cells expressing control DARPin E3_5* (not interacting with LmrCD) (Δ). (**B**) No differences were observed when experiments in (A) were performed with cells lacking the chromosomal copy of *lmrCD*. (**C**) BCECF-AM transport measurements in pre-energized wildtype *L. lactis* cells demonstrate activation of LmrCD-mediated extrusion upon expression of DARPin_Act2 (trace 1) but not of control DARPin E3_5* (trace 2). No activation of LmrCD activity was observed upon expression of DARPin_Act2 (trace 3) or control DARPin E3_5* (trace 4) in *L. lactis* Δ*lmrCD* cells. Shown are representative data from at least three independent measurements (n≥3).

LmrCD-mediated daunomycin resistance in *L. lactis* [53] was used for screening of DARPins that affect LmrCD activity. Individual DARPins of the pool obtained after four selection rounds (Figure 2; note: these are not the DARPin binders identified by ELISA from the previous section) were expressed at high levels in the cytoplasm of *L. lactis* using the nisin-inducible lactococcal vector pNZ8048 (estimated to 2–5% of total soluble protein, not shown) [54]. We first attempted to find DARPins whose expression lead to a decrease of LmrCD-dependent daunomycin resistance (inhibitors). Around 20 apparent inhibitors were found by screening 400 DARPin clones expressed in *L. lactis*. A closer inspection of these initial hits however, revealed that they were false positives; lactococcal cells expressing these DARPins grew considerably slower than cells expressing the control DARPin E3_5*. When these DARPin inhibitors were expressed in the *L. lactis* strain lacking the chromosomal *lmrCD* genes (*L. lactis* NZ9000 Δ*lmrA* Δ*lmrCD* [55]), the apparent inhibition was also observed. Hence, the increased drug susceptibility of *L. lactis* expressing these DARPins was independent of LmrCD. Surprisingly, we also found DARPins the expression of which increased daunomycin resistance in *L. lactis*, suggesting enhancement of LmrCD activity. Three strong activators (DARPin_Act1-3) were found in a screen including 1128 clones (Figure 2). In cell growth experiments, the daunomycin resistance of *L. lactis* NZ9000 expressing the activator DARPins was compared to the control DARPin E3_5* in the wildtype and the Δ*lmrCD* background (Figure 4A and B). In wildtype cells, the IC_50_ for daunomycin was increased by a factor of 3.3, 2.6 and 1.7 upon the production of DARPin_Act1, DARPin_Act2, and DARPin_Act3, respectively. Importantly, the expression of the activator DARPins in the *L.* *lactis* NZ9000 Δ*lmrA* Δ*lmrCD* background did not affect the daunomycin resistance of the cells, indicating an LmrCD-specific functional stimulation. The knock-out of *lmrCD* in *L. lactis* results in an 8.3-fold decrease of the IC_50_ for daunomycin (Figure 4A and B). Therefore, the DARPin-induced stimulation of LmrCD-mediated drug transport by a factor up to 3.2 is substantial. The DARPins α-LmrCD#1-5 that were identified in the ELISA screen to bind to LmrCD (see previous section) were also assayed regarding the potential modulation of the LmrCD-mediated drug resistance in *L. lactis*. Although DARPins α-LmrCD#1-5, the DARPin activators and DARPin E3_5* were overproduced equally well in *L. lactis*, expression of DARPins α-LmrCD#1-5 did not alter the drug resistance of lactococcal cells towards daunomycin (not shown).

### Further Characterization of the LmrCD-activating DARPins

The chromosomal knock-out of the *lmrCD* gene on *L. lactis* has been shown to result in an increased susceptibility of the lactococcal cells towards Hoechst 33342 (3.6 fold difference between wiltype *L. lactis* and the Δ*lmrCD* mutant) [53]. We therefore tested whether the DARPin_Act1 to Act3 are also capable of increasing the LmrCD-mediated transport of Hoechst 33342. However, in contrast to the observations on daunomycin resistance in *L. lactis* (Figure 4) the expression of the DARPin activators did not increase the resistance towards Hoechst 33342 (not shown).

The observed LmrCD-associated daunomycin resistance in *L.* *lactis* could be due to enhanced drug efflux by LmrCD. However, as the entry of fluorescent daunomycin from the aqueous buffer into cells followed by its intercalation in DNA results in a minor quenching of total fluorescence, detection of daunomycin transport by fluorescence spectroscopy is hampered by a poor signal-to-noise ratio in the fluorescence data. In an alternative assay, we studied the LmrCD-mediated transport of non-fluorescent, hydrophobic 2′,7′-bis-(2-carboxyethyl)-5(6)-carboxyfluorescein acetoxymethyl ester (BCECF-AM), which is extruded from the plasma membrane by bacterial and mammalian multidrug ABC transporters before it can be hydrolyzed in the cytoplasm into fluorescent BCECF by non-specific esterases [56]–[58]. In this assay, a slower increase in the fluorescence signal is associated with enhanced extrusion of BCECF-AM from the cell. As BCECF is a pH-sensitive fluorophore, valinomycin and nigericin were added to the cells prior to the transport measurement to dissipate the electrochemical proton gradient across the plasma membrane, so that the intracellular pH was made equal to the constant pH of the extracellular buffer. In agreement with the observations for daunomycin, increased BCECF-AM efflux was observed upon expression of DARPin_Act2 in wildtype *L. lactis*, whereas DARPin_Act2 expression in the *lmrCD* knockout-strain did not affect transport (Figure 4C).

Sequencing revealed that DARPin_Act1 lacked the N-terminal cap repeat and therefore exhibited severe aggregation (but not precipitation) in purified form as demonstrated in SEC experiments (Table 1, Figure S1D). This impeded further biochemical and biophysical characterization of DARPin_Act1. Although DARPin_Act2 was of the expected N3C format, it was prone to form soluble aggregates (hexamers), presumably due to a high number of hydrophobic residues found in its randomized positions. DARPin_Act3 predominantly existed as a monomer and the aggregated species could successfully be removed by SEC.

### Expression of LmrCD-activating DARPins does not Increase the LmrCD Production Level

**Figure 5:**
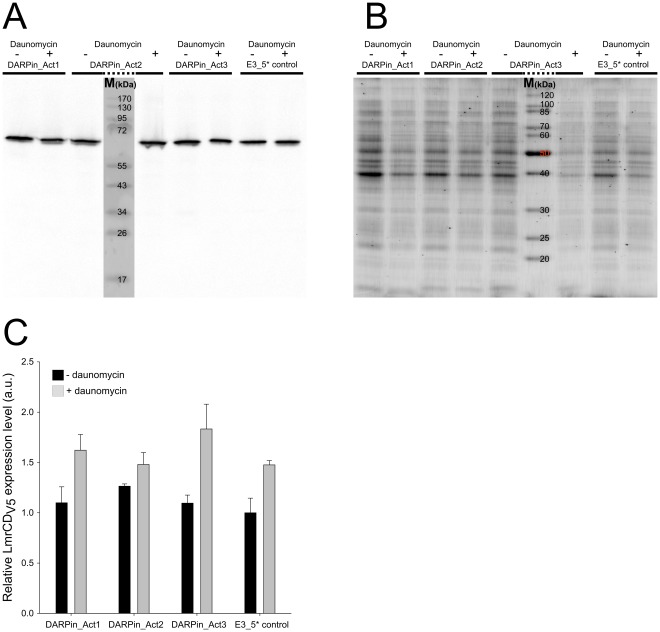
DARPin expression does not significantly alter expression of LmrCD proteins. (**A**, **B**) A V5-tag was introduced in frame at the 5′-end of genomic *lmrD* in *L. lactis* (denoted *L. lactis NZ9000 lmrD_V5_*). Plasmid-encoded DARPin activators or the control DARPin E3_5* were expressed in *L. lactis NZ9000 lmrD_V5_* in the presence and absence of daunomycin (14 µM for DARPin_Act3 and E3_5* and 28 µM for DARPin_Act1 and DARPin_Act2, respectively). The expression levels of genomic LmrD_V5_ were then quantified by comparing the Western blot signal obtained using an anti-V5 antibody (A) with total protein detected by SYPRO ruby staining (B). (**C**) The relative amounts of LmrD_V5_ expression were quantified by densitometry. Each bar represents the average of three independent data points (n = 3) of which one data point is shown in (A) and (B).

The observed gain of cellular drug resistance and enhanced rates of substrate efflux in DARPin producing cells could be explained if DARPin expression would upregulate the expression level of LmrCD. In order to compare the amounts of expressed LmrCD protein in the plasma membrane from DARPin-producing and control cells, we introduced a V5-tag downstream to the *lmrD* copy on the chromosome by homologous recombination. Cells producing the V5-tagged version of LmrD (LmrD_V5_) were as resistant to daunomycin as the wildtype cells. A specific band for LmrD_V5_ could be detected by Western blotting with an anti-V5 antibody (Figure 5A). *L. lactis* NZ9000 *lmrD_V5_* expressing the activator DARPins and the control DARPin E3_5* were grown in the absence of drug and in the presence of the daunomycin concentration (see Materials and Methods). The amount of LmrD_V5_ was then analyzed by Western blotting whereas the total protein was quantified using SYPRO ruby staining. The LmrD_V5_ production level was consistently increased by a factor of around 1.5 upon the exposure to daunomycin irrespective of the DARPin expressed (Figure 5B). However, the activator DARPins did not lead to a significant increase in LmrD_V5_ production compared to the control cells, indicating that the DARPin activators directly stimulate the drug efflux activity of existing transporters.

### LmrCD-specific DARPins Bind to LmrD in a 1∶1 Stoichiometry Covering at least Three Partially Overlapping Epitopes

To gain further insights into the binding epitopes of the LmrCD-specific DARPins, LmrC and LmrD were expressed separately including GFP fused to the C-termini. The proteins were purified by Ni^2+^-NTA chromatography, followed by chemical biotinylation and size exclusion chromatography (Figure S1E). Further, the isolated NBDs of LmrC and LmrD were purified from *E. coli*. Whereas the expression of the NBD of LmrC gave rise to soluble aggregates exclusively (which were not used for further analysis), purification of the NBD of LmrD yielded (besides soluble aggregates) monomeric protein that was enzymatically biotinylated (bLmrD-NBD_AviN_) (Figure S1F). Binding of these isolated parts of LmrCD to α-LmrCD#1-5 and the activator DARPins was then tested in an ELISA (Figure 3B). All DARPins were found to bind to the LmrD chain, but none of them recognized the NBD of LmrD or the LmrC chain suggesting that the epitope(s) are likely to be located at the membrane domain of LmrD. Alternatively, the isolated NBD of LmrD might adopt a conformation different to the one found in the full-length transporter which might not be recognized by the DARPins or the binding epitope covers a shared surface located on the MD and the NBD of LmrD.

**Figure 6:**
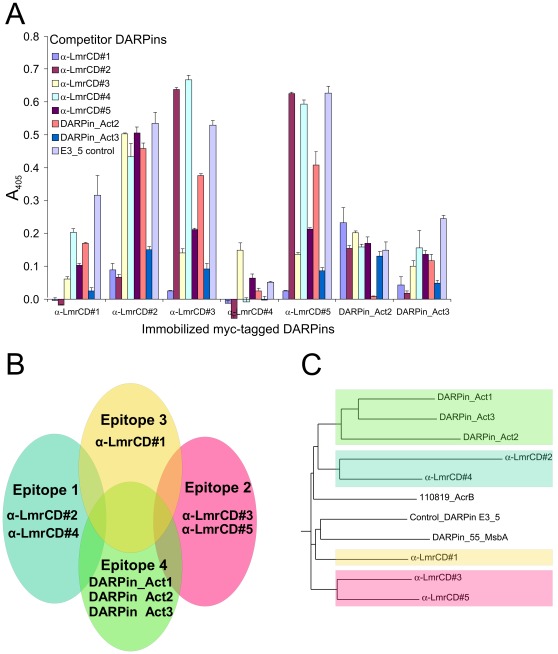
Epitope mapping of LmrCD-specific DARPins by ELISA. (**A**) Analysis of the LmrCD-specific DARPins by a competition ELISA. Binding of bLmrCD_AviC_ to immobilized Myc-tagged DARPins was competed with an excess of DARPins devoid of Myc-tag. (**B**) Schematic drawing of the four proposed binding epitopes on LmrCD recognized by the LmrCD-selective DARPins based on the results of the competition ELISA shown in (A). The number of the epitopes follows the numbering in the main text. (**C**) The phylogenetic tree of the LmrCD-specific DARPins corresponds well with the proposed binding epitopes. The branches of the phylogenetic tree are highlighted with the color code used to label the four suggested binding epitopes in (B).

The binding epitopes were further analyzed in a competition ELISA, in which bLmrCD_AviC_ was pre-incubated with a tenfold excess of each DARPin devoid of the Myc-tag and probed for binding to every DARPin_myc5_ immobilized via the Myc-tag (Figure 6A). Based on the results of this competition ELISA, the LmrCD-specific DARPins are proposed to bind to at least three partially overlapping epitopes (Figure 6B). The first epitope (epitope 1 of binders α-LmrCD#2 and α-LmrCD#4) and the second epitope (epitope 2 of binders α-LmrCD#3 and α-LmrCD#5) do not overlap (i.e. no competition for binding between these two pairs of DARPins to LmrCD was observed). In contrast, binding of α-LmrCD#1 and the DARPin activators (DARPin_Act2 and DARPin_Act3) to LmrCD is competed by DARPins recognizing epitopes 1 and 2 as well as by themselves. Hence the binding epitopes of α-LmrCD#1 and the DARPin activators are suggested to partially overlap with the first two epitopes. Nevertheless, conformational communication between two well-separated sites resulting in apparent competition of binding cannot be excluded. The presence of two distinct epitopes, one for α-LmrCD#1 (epitope 3) and the other for activator DARPins (epitope 4), is supported by the large differences in sequence between α-LmrCD#1 and the activator DARPins (Figure 6B, Figure S2). Trapping of LmrCD with vanadate did not help to generate DARPins different from the ones selected in the absence of trapping agent as DARPin α-LmrCD#2 (non-vanadate DARPin) shares the epitope with α-LmrCD#4 (vanadate DARPin), and α-LmrCD#5 (non-vanadate DARPin) shares the epitope with α-LmrCD#3 (vanadate DARPin). Clearly, “vanadate” DARPins do not bind to a shared epitope that is distinct from the epitope of the “non-vanadate” binders.

**Figure 7:**
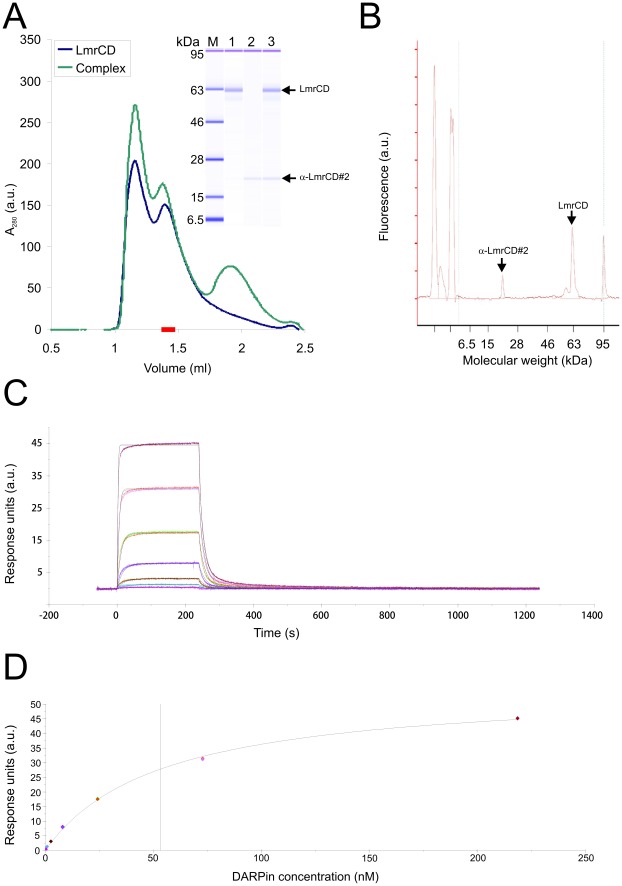
Biophysical characterization of the DARPin-LmrCD complexes. (**A**, **B**) Stoichiometry analysis as exemplified by the LmrCD/α-LmrCD#2 complex. (A) LmrCD and the LmrCD/α-LmrCD#2 complex were separated by SEC (Superdex 200 PC3.2/30, GE Healthcare) with a void volume V_0_ = 0.85 ml and a total volume V_t_  = 2.4 ml. A fraction corresponding to heterodimeric LmrCD in complex with α-LmrCD#2 complex (red bar) was subjected to protein chip analysis (lane 3, inset). LmrCD and the DARPin α-LmrCD#2 were also analyzed (lanes 1 and 2, inset). The peak at a retention volume of 1.2 ml corresponds to aggregated LmrCD. (B) The peak area of the protein chip chromatogram corresponding to LmrCD and α-LmrCD#2 of lane 3 in (A) were calibrated with dilution series of LmrCD and DARPin of known protein concentrations (not shown) and were used to determine the stoichiometry of the LmrCD-DARPin complexes (Table 1). (**C**) Affinities of the DARPins to LmrCD were determined by surface plasmon resonance as shown for α-LmrCD#3. The colored lines correspond to the measured traces at different DARPin concentrations, the fitted curves (1∶1 binding model) are shown as black lines. (**D**) The steady state DARPin binding signals achieved at the end of the association phase shown in (C) were plotted against the DARPin concentration and fitted using an equilibrium binding equation equivalent to the Michaelis-Menten equation. In this analysis, equilibrium dissociation constants (*K*_D, eq._) were generated.

The stoichiometry of binding between the DARPins and LmrCD after SEC was determined by protein chip technology (Agilent Technologies) allowing accurate quantification of protein amounts (Figure 7A and B, Table 1). DARPin_Act3 as well as α-LmrCD#1, α-LmrCD#2, α-LmrCD#3 and α-LmrCD#5 form 1∶1 complexes with LmrCD (Table 1). DARPin_Act1 and DARPin_Act2 formed soluble aggregates impeding their separation from LmrCD on SEC whereas the affinity of α-LmrCD#4 appeared to be too low to allow co-elution with LmrCD from the gel filtration column. Therefore, the stoichiometry of binding could not be determined for these DARPins.

### Determination of the Dissociation Constants by Surface Plasmon Resonance (SPR)

The affinities of the isolated DARPins to LmrCD were determined by SPR measurements using a Biacore instrument. Detergent purified bLmrCD_AviC_ was immobilized on a streptavidin-coated chip and binding of the DARPins was assessed (Figure 7C and D, Table 1). When using a two-state reaction model (see Materials and Methods), the observed data fitted very close to the predicted data. However, to assess whether DARPin binding to LmrCD is correctly described by a two-state reaction model, 400 nM of α-LmrCD#3 was injected for 100 s, 200 s and 400 s, and DARPin dissociation phases were compared (Figure S3). The dissociation curves obtained, superimposed almost perfectly, suggesting that DARPin dissociation was independent of the association time. These findings indicate that the use of the two-state reaction model is inappropriate. Therefore, all data were fitted using a simple 1∶1 binding model (see Materials and Methods), which allowed for the calculation of the dissociation constants (*K*_D_) from the association and dissociation rate constants k_a_ and k_d_ (Table 1). To determine equilibrium binding constants (*K*_D,eq._, see Materials and Methods, Figure 7D and Table 1), injection times were chosen that allowed DARPin binding to reach equilibrium (Figure 7C). With the exception of α-LmrCD#2 and DARPin_Act2, the *K*_D_ and *K*_D,eq._ were found to be almost identical. Since *K*_D,eq._ is unaffected by known SPR artifacts such as mass transport and analyte rebinding [59], we refer to the *K*_D,eq._ to describe the affinities of the DARPins for LmrCD in this study. The *K*_D,eq._ values of the majority of LmrCD-specific DARPins were between 9 nM and 67 nM with the exception of the *K*_D,eq._ of 173 nM for α-LmrCD#4. Confirming the SPR measurements, α-LmrCD#4 binding to LmrCD was too weak for co-elution of the protein complex during SEC (Table 1); the ELISA signal was considerably lower than for the other binders (Figure 3A).

### DARPin Binding to Membrane-embedded LmrCD

**Figure 8:**
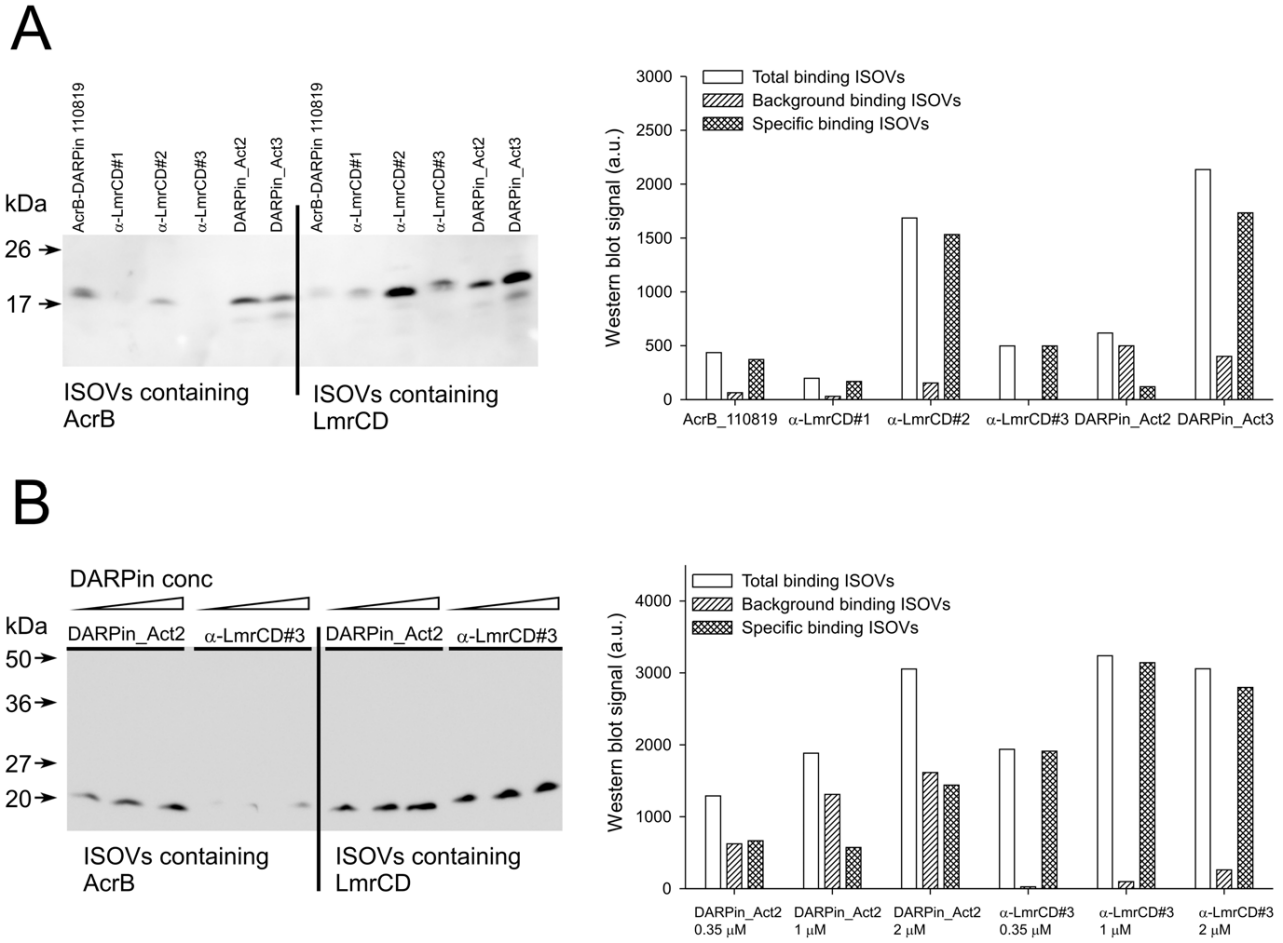
DARPin binding to membrane-embedded LmrCD. (**A**) Six DARPins (each at a 350 nM concentration) specific for AcrB or LmrCD were probed for binding to ISOVs containing either overproduced AcrB_AviC_ or LmrCD_AviC_. Bound DARPins were detected on Western blot (left panel). The signals of the DARPin-specific bands were quantified by densitometry (right panel). Total binding denotes the quantified amount of DARPin bound to membrane vesicles containing overexpressed target protein. Background binding refers to binding to membrane vesicles containing overexpressed LmrCD_AviC_ in case of the AcrB DARPin 110819, or overexpressed AcrB_AviC_ when LmrCD-specific DARPins were used. Specific binding was calculated by subtracting background binding from total binding. (**B**) Binding of DARPin_Act2 and α-LmrCD#3 to ISOVs containing either overproduced AcrB_AviC_ or LmrCD_AviC_ was further assessed using increasing concentrations of DARPin (0.35 µM, 1 µM and 2 µM) and analyzed by Western blot (left panel). The data was quantified as in (A) (right panel). The data represent typical results observed in n = 3 experiments.

The binding of DARPins to inside-out membrane vesicles (ISOVs) containing either overproduced AcrB_AviC_ or LmrCD_AviC_ was further characterized (Figure 8). Based on an analysis using a protease-cleavable LmrCD-GFP construct (see Materials and Methods), ISOV preparations were found to contain up to 10% of the membrane vesicles in the right-side-out orientation (right-side-out membrane vesicles, RSOVs). Total binding was determined as the amount of DARPin bound to ISOVs containing the overexpressed target protein. Background binding refers to binding of the respective DARPin to ISOVs containing an overexpressed membrane protein that is not recognized by the binder. For the AcrB-specific DARPin 110819, the membrane vesicles used for the determination of background binding thus contained overexpressed LmrCD and *vice versa*. Specific binding was then calculated by subtracting background binding from total binding. Binding of all six DARPins tested was target-specific, meaning that total binding was stronger than background binding. The AcrB-specific DARPin 110819, whose structure has been solved in complex with AcrB by X-ray crystallography, was used as control. As expected, DARPin 110819 binds relatively poorly to ISOVs despite its high reported binding affinity of 28 nM because the binding epitope on AcrB is located at the periplasmic loops and is therefore predominantly hidden in the vesicle lumen [18]. The binding signal for DARPin 110819 therefore originates from the estimated 10% RSOVs present in the ISOV preparation. Despite the fact that AcrB is expressed better than LmrCD (not shown), binding of α-LmrCD#2 and DARPin_Act3 to LmrCD-containing ISOVs resulted in signals that were around three times bigger than the ones of DARPin 110819 binding to AcrB-containing ISOVs (Figure 8A). Since the binding affinities of α-LmrCD#2 (9 nM) and DARPin_Act3 (55 nM) are in the same order of magnitude as of DARPin 110819 (28 nM), these LmrCD-specific DARPins appear to recognize epitopes at the cytoplasmic portion of LmrD, which are accessible in ISOVs. Specific binding of α-LmrCD#1 on the other hand is half as high as for DARPin 110819 whereas it is roughly the same for α-LmrCD#3. DARPin binding to these epitopes is therefore either restricted in membrane-embedded LmrCD or the epitope is only accessible from the physiological outside of the membrane. We also attempted to perform these DARPin binding experiments using RSOVs generated from *E. coli* using the EDTA-lysozyme method [60]. Studies on the accessibility of a C-terminal GFP fusion partner on LmrD to protease cleavage from the external surface of membrane vesicles indicated that, despite careful preparations, a substantial portion (up to 50%) of LmrCD-GFP containing membrane vesicles were in the inside-out orientation, and that therefore, this type of membrane vesicles could not be used to study the accessibility of the binding epitopes (data not shown). Background binding to ISOVs varied between the different DARPins and correlated with the aggregation behavior on SEC (Figure S1). Low background binding was observed for the DARPins α-LmrCD#1, α-LmrCD#3 and the AcrB-DARPin 110819, whereas α-LmrCD#2, DARPin_Act2 and DARPin_Act3 interacted with membrane vesicles lacking the target protein (Figure 8A).

Specific binding of DARPin_Act2 to LmrCD in ISOVs was low in the initial binding experiment, most likely due to its slow on-rate of binding (Figure 8A, Table 1). Therefore, binding of DARPin_Act2 and α-LmrCD#3 to membrane-embedded LmrCD in ISOVs was determined at a prolonged incubation time (200 min instead of 40 min) and at increasing DARPin concentrations (0.35 µM as in the initial experiment, 1 µM and 2 µM) (Figure 8B). Although background binding of DARPin_Act2 remains high, specific binding was substantially increased, in particular at a DARPin concentration of 2 µM. For the DARPin α-LmrCD#3 on the other hand, background binding was very low and maximal specific binding was achieved already at a concentration of 1 µM. Taken together, these binding assays suggest that specific protein-protein interactions between the activator DARPins and membrane-embedded LmrCD are likely to provide the basis for the activation of LmrCD-mediated drug transport, although indirect mechanisms due to binding of the DARPin activators to the membrane cannot be excluded. Binding of DARPin_Act2 and DARPin_Act3 to LmrCD-containing ISOVs indicates that the DARPin activators can bind to their epitope on LmrD when expressed in the cytoplasm of *L. lactis*. If we assume a protein concentration of 200 mg/ml in the cytoplasm of *L. lactis* [61] and estimate the DARPin expression level to amount for 2% of total protein (not shown), the DARPin concentration inside the cell is about 4 mg/ml or 200 µM. The DARPin concentration in the cell exceeds its binding affinities by more than three orders of magnitude and therefore the binding epitopes are saturated with bound DARPins.

### DARPin Activators Stimulate the Basal ATPase Activity of LmrCD Reconstituted in Proteoliposomes

**Figure 9:**
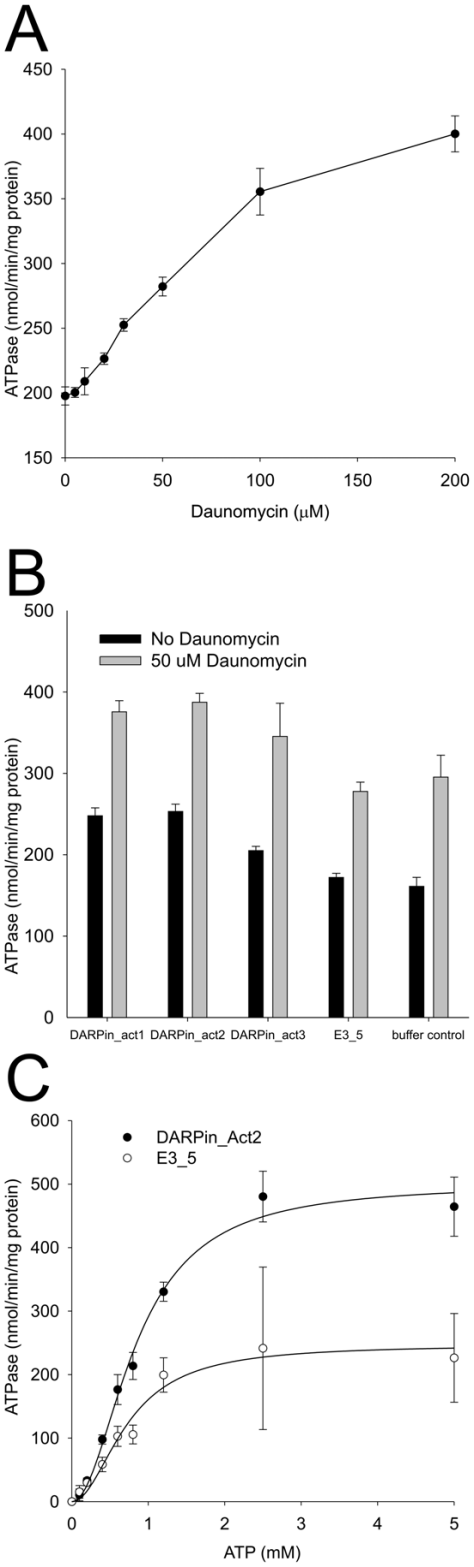
ATPase activity of reconstituted LmrCD is stimulated by DARPin activators and daunomycin. Each symbol or bar represents the average of three data points. (**A**) The ATPase activity of reconstituted LmrCD is stimulated in the presence of daunomycin in a dose-dependent manner. (**B**) Reconstituted LmrCD (protein:lipid ratio of 1∶50, proteoliposomes diluted to obtain an LmrCD concentration of 70 nM) was incubated with DARPin activators and control DARPin E3_5* (2.5 µM) and the ATPase activity was determined in the absence and presence of 50 µM daunomycin (triplicates). As a control, buffer instead of DARPins were added to LmrCD. According to t-test analysis, the measured ATPase activity differences between DARPin_Act1 to Act3 and the buffer control are statistically significant (p<0.01 in the absence and p<0.05 in the presence of daunomycin, respectively). (**C**) The ATPase activities of LmrCD in the presence of DARPin_Act2 and E3_5 were determined over a range of ATP concentrations. The data points were fitted to the Hill equation.

To further elucidate the mechanism by which the DARPin activators stimulate the function of LmrCD, detergent-purified LmrCD was reconstituted into proteoliposomes made of polar *E.* *coli* lipids and egg-phosphatidylcholine mixed at a ratio of 3∶1 [55]. Reconstituted LmrCD exhibits basal ATPase activities that are three times lower than the activity of purified LmrCD in its micellar form (not shown). Addition of increasing concentrations of daunomycin to reconstituted LmrCD (5–200 µM) increased its ATPase activity in a dose-dependent manner, reaching two-fold stimulation at 200 µM daunomycin (Figure 9A). The ATPase activity of reconstituted LmrCD in the presence of the DARPin activators and the control DARPin E3_5 was then compared to samples to which no DARPins were added (Figure 9B). The addition of DARPin E3_5 did not change the ATPase activity of LmrCD at any concentration of daunomycin. On the other hand, ATP hydrolysis of LmrCD was significantly stimulated upon addition of the three DARPin activators up to 1.6 fold in case of DARPin_Act2. These observations in proteoliposomes were found to be statistically significant in three independent reconstitution experiments, one of which is shown in Figure 9B. The DARPin activators are therefore capable of increasing the ATPase activity of LmrCD to a similar extent as 50 µM of daunomycin for which a 1.8 fold increase is seen (Figure 9A and B). The increase of LmrCD’s ATPase activity by the DARPin activators and daunomycin was found to be additive, suggesting that the molecular mechanism underlying these stimulatory effects are distinct. Basal and DARPin_Act2-stimulated ATPase activity of reconstituted LmrCD was further elucidated over a range of ATP concentrations (Figure 9C). The data was fitted using the Hill equation, and the apparent *K*_m_ for ATP and V_max_ of the ATPase reaction as well as the Hill coefficient were determined. The errors represent standard errors of the parameters derived from nonlinear regression analysis. In presence of DARPin_Act2, the apparent affinity of LmrCD for ATP was not significantly altered (*K*_m,app_ of 0.85±0.06 mM and 0.73±0.09 mM for DARPin_Act2 and E3_5, respectively). V_max_ on the other hand was doubled in the presence of DARPin_Act2 (V_max_ of 500±22 nmol/min/mg of protein versus 247±19 nmol/min/mg of protein). The Hill coefficient was found to be unaltered in presence of DARPin_Act2 (2.0±0.3 and 2.0±0.5 for DARPin_Act2 and E3_5, respectively). The sigmoidal nature of the fitted curve suggests positive cooperativity between the non-canonical and the consensus composite catalytic site of LmrCD, a finding reminiscent of the maltose transporter and the isolated NBDs of HlyB [62], [63].

## Discussion

The *in vitro* selection of binders against integral membrane proteins using ribosomal display is very fast (2–3 weeks of lab work under ideal circumstances) and the biochemical conditions can be controlled. Nevertheless, only few successful examples of *in vitro* selected binders specific for membrane protein have been reported, most likely due to the many unknowns that exist regarding enrichment of specific binders against these hydrophobic proteins [18], [29]–[36]. In this work we have made important progress in the screening procedure of DARPins raised against membrane proteins. We found that successful *in vitro* selection depends on two critical factors. Firstly, the quality of the target protein preparation is crucially important for success. LmrCD has proven to be a suitable target since it could be purified to near homogeneity, was catalytically active and could be isolated as heterodimeric species by SEC (Figure S1A and B). Secondly, during DARPin identification it is important to introduce a cross-specificity ELISA using a set of different membrane proteins. Using optimally prepared LmrCD, we obtained a relatively large number of DARPins (around 70%) that showed strong cross-reactivity with MsbA and AcrB. Further analysis showed that many of these unspecific DARPins formed soluble aggregates. Both observations might relate to the hydrophobicity of the target proteins, which can drive selection of hydrophobic binding surfaces in DARPins that tend to aggregate in an aqueous environment. Indeed, DARPin aggregation was not observed at all when DARPins were selected against a soluble test protein (MBP) using the same selection procedure (data not shown). On the other hand, the highly specific DARPins were much less aggregation-prone and about half of them were monomeric as judged from comparing the SEC profiles of the DARPins under study with the monomeric control DARPin E3_5 (Figure S1C). Because the cross-specificity ELISA was performed with DARPin-containing crude cell extracts, there was no need to purify the DARPins for the initial specificity analysis, which greatly accelerated the identification of binders. This screening regime would also be applicable to more difficult membrane protein targets with a further decreased binder hit rate.

A handful of the LmrCD-specific DARPin binders were subsequently characterized by surface plasmon resonance and size exclusion chromatography. With the exception of the DARPin α-LmrCD#4, the *K*_D,eq._ values for binding were found to range between 9 and 67 nM. The binding stoichiometry of these high-affinity binders with heterodimeric LmrCD is 1∶1. The LmrCD-specific DARPins are suggested to recognize at least three overlapping epitopes on the LmrD chain. The surface of LmrD might therefore harbor one or several hot spot epitopes that are preferably recognized by the DARPins. Recently, a hot spot epitope that is recognized by nine highly diverse DARPins has been reported for AcrB [19]. The fact that a handful of high quality DARPins specific for LmrCD could be readily identified, indicates that the randomized DARPin scaffold is sufficiently diverse to recognize a multitude of binding sites on the membrane protein target. Given the high binding affinities achieved and the various epitopes recognized on LmrCD, these DARPins can be used for chaperone-assisted membrane protein crystallography [64].

Binding experiments using LmrCD-containing ISOVs suggest that the DARPins α-LmrCD#2 and DARPin_Act3 bind to epitopes located at the cytoplasmic side of LmrCD. DARPin activators expressed in the *L. lactis* cells are therefore expected to readily reach their binding epitope *in vivo*. Since these DARPins recognize full-length LmrD, but not the isolated NBD of LmrD, it is likely that they bind to the cytoplasmic loops of the membrane domain of LmrD. The other LmrCD-specific DARPins tested (α-LmrCD#1 and α-LmrCD#3) were found to bind to membrane-embedded LmrCD as well. However, the relatively weak binding signals suggest that access to the binding epitopes is either partially restricted by the lipid bilayer or that the binding epitope is only accessible from the physiological outside of the cell, which is hidden in the vesicle lumen of ISOVs. In the latter case, the binding signal would originate from the approximate 10% of RSOVs found in ISOV preparations.

The drug resistance phenotype in *L. lactis* associated with the genomic expression of LmrCD was used to screen our pre-selected DARPins for those that influence the functional properties of this multidrug transporter. We observed the production of DARPins in the cytoplasm of *L. lactis* with a relatively low toxicity compared to expression in *E. coli*. Three homologous DARPins (DARPin_Act1, Act_2, and Act3) were obtained, which enhance the LmrCD-associated resistance to daunomycin and activate efflux of BCECF-AM, but which, surprisingly, do not alter the resistance to Hoechst 33342. This finding is reminiscent to a study on ABCB1, in which small molecules were found to increase its transport activity for some drugs whereas the transport of other drugs was not affected or even decreased [65].

We considered the possibility of an increased LmrCD production level in *L. lactis* in the presence of DARPin activators that might act as folding chaperones. To test this hypothesis, a V5-tag was introduced in frame with *lmrD* on the chromosome of *L. lactis*, an approach that, to the best of our knowledge, was carried out for the first time in this bacterium. With this tool it was demonstrated that the expression of the activator DARPins in *L. lactis* does not lead to changes in LmrCD production levels in the presence as well as in the absence of daunomycin. As a proof of concept, LmrCD expression was increased 1.5-fold in the presence of daunomycin, which agrees well with RT-PCR experiments detecting a transient two-fold increase of mRNA transcription from *lmrCD* upon drug stimulation [66]. From this experiment we concluded that the increased daunomycin resistance as well as the enhanced BCECF-AM efflux originates from a direct stimulation of the activity of LmrCD transporters as a consequence of DARPin binding.

To gain more insight into the potential mechanism underlying the activation of drug transport, the influence of the DARPin activators on the ATPase activity of reconstituted LmrCD was studied. The DARPin activators were found to stimulate the ATPase activity of reconstituted LmrCD to a similar extent as daunomycin applied at a concentration of 50 µM. Activation of the basal ATPase activity of LmrCD upon DARPin expression is a plausible explanation for the observed daunomycin resistance increase in *L. lactis*. However, it cannot explain why the resistance of lactococcal cells to Hoechst 33342 was not affected by the expression of the DARPin activators. The exact mechanism behind the modulation of LmrCD-mediated drug transport by the DARPin activators is possibly much more complex. Recent studies on Pdr5, a heterodimeric multidrug transporter of *Saccharomyces cerevisiae* revealed a single mutation at one NBD which abolished drug resistance against rhodamine-like compounds whereas transport of other drugs was unaffected [67]. Likewise, a screen identified small molecules dramatically altering the drug transport profile of ABCB1 based on a molecular mechanism that remains elusive [65]. These findings cannot yet be comprehensively explained by current models of ABC transporter mechanism and illustrate the limitation of our knowledge.

Beyond the activation of the basal ATPase by the DARPin activators, we speculate that DARPin binding to LmrCD might stabilize a conformational transition state at a rate-limiting step during daunomycin and BCECF-AM transport. DARPin binding could, for example, increase the overall rate of transport by stabilizing the inward-facing state resulting in increased fractional occupation during substrate binding, or enhance the dissociation of the substrate from outward-facing LmrCD. But also the resetting of LmrCD from the outward-facing to the inward-facing state after ATP hydrolysis and drug release might be accelerated by the DARPin activators. Finally, in addition to these possible effects of DARPin binding on the maximal rate of efflux, DARPin binding might directly influence the drug binding affinity of LmrCD by imposing structural changes in drug binding surfaces. The effect of DARPins on the mechanism of transport in *in vitro* models (e.g. proteoliposomes) will be studied in future work.

In conclusion, we obtained three DARPins that activate multidrug export by LmrCD in intact cells and stimulate the ATPase activity of the transporter reconstituted into proteoliposomes. Our work demonstrates the potential of *in vitro* selected artificial binding molecules to manipulate membrane transport processes *in vivo*. Unlike chemical modulators, binding proteins have the potential to stabilize any conformational (transition) state of a membrane transporter, and offer the possibility to functionally and structurally study membrane proteins in unprecedented ways. When targeting membrane transporters associated with human disease, DARPins could therefore be of great biopharmaceutical importance.

## Materials and Methods

### Molecular Cloning and Expression of lmrCD and Other Transporters

The primers and genetic constructs are listed in Table S1 and Table S2. The *lmrCD* genes as well as the genes of *msbA* and *acrB* were cloned with a coding region for an Avi-tag sequence at their 3′-end, which allows the site-specific biotinylation of the target proteins for the purpose of protein immobilization during ribosome display and ELISA. A DNA fragment encoding the Avi-tag sequence flanked by the restriction sites NheI and BamHI was formed by annealing the two oligonucleotides avitag_for and avitag_rev, and was ligated into the *E. coli* cloning vector pGEM using the NcoI and XbaI restriction sites, yielding pGEM_Avi. The *lmrCD* genes were amplified from the chromosome of *Lactococcus lactis* subsp. *cremoris* MG1363 using the primers lmrCD_DecaHisN_AviC_for for introduction of an N-terminal His10-tag in LmrC and lmrCD_AviC_rev to add a C-terminal Avi-tag to LmrD. The PCR product was cut with NcoI and XbaI and cloned into the pGEM_Avi digested with NcoI and NheI yielding pGEMLmrCD_AviC_. Two independent clones were sequenced and were found to carry a nucleotide substitution compared to the published sequence of *Lactococcus lactis* subsp. *cremoris* MG1363 [68] at the triplet position of C179 in LmrC, which is an arginine in our clone (TGC → CGC). In addition, a construct lacking the C-terminal Avi-tag was cloned by amplifying *lmrCD* from pGEMLmrCD_AviC_ using the forward primer lmrCD_NdeI_Presc_that introduces a linker and a prescission protease cleavage site at the 5′-end, and the reverse primer lmrCD_rev. The PCR product was digested using NdeI/XbaI and ligated into pGEMLmrCD_AviC_ cut with the same enzymes, resulting in plasmid pGEMLmrCD. The tagged *lmrCD* genes were then sub-cloned via NcoI/XbaI either into the lactococcal pNZ8048 vector [54] or the *Escherichia coli* expression vector pBAD24 [69] yielding the expression vectors pNZLmrCD_AviC_, pNZLmrCD, pBADLmrCD_AviC_ and pBADLmrCD, respectively. The *msbA* gene was cloned into pGEM_Avi via the restriction sites NcoI/NheI amplifying the *msbA* gene with the primers msbA_DecaHisN_for and msbA_AviC_rev from the clone pNZMsbA [44] yielding pGEMMsbA_AviC_. The gene of *acrB* from *E. coli* devoid of NcoI sites (Murakami and van Veen, unpublished) was amplified with the primers acrB_HisC_AviC_for and acrB_HisC_AviC_rev and cloned via NcoI/NheI into pGEM_AviC yielding pGEMAcrB_AviC_. The tagged *msbA* and *acrB* genes were sub-cloned into pBAD24 using the restriction sites NcoI and XbaI resulting in pBADMsbA_AviC_ and pBADAcrB_AviC_. All sequences were confirmed by DNA sequencing. The genes coding for *lmrC*, *lmrD* and *lmrCD* were also cloned in frame with a C-terminal GFP (that is cleavable by 3C protease) into pBAD24 applying the recently developed FX-cloning method [70]. Similarly, coding regions of the NBDs of LmrC and LmrD (which includes residues G336 to D579 and G424 to E664 of LmrC and LmrD, respectively) were cloned into a FX-vector adding a His10-tag, a 3C protease cleavage site and an Avi-tag to the 5′-end of the cloned genes (Geertsma and Dutzler, unpublished). The Walker B glutamate of the consensus ATPase site of LmrCD was mutated to glutamine using a quick-change standard protocol (LmrD_E587Q). LmrCD protein containing a C-terminal Avi-tag (LmrCD_AviC_) was produced in and purified from *L. lactis* NZ9000 Δ*lmrA* Δ*lmrCD* [55] following published protocols [45], [71]. The enzymatic site-specific biotinylation of the Avi-tag was carried out *in vitro* using purified BirA yielding biotinylated LmrCD_AviC_ (bLmrCD_AviC_) [51], which was then used for DARPin selection and ELISA. MsbA_AviC_ and AcrB_AviC_ were expressed in *E. coli* harboring the corresponding pBAD24 expression vectors and were purified and biotinylated accordingly.

### DARPin Selection

The N3C DARPin library was chosen to select binders against biotinylated LmrCD_AviC_ (bLmrCD_AviC_) using the ribosome display method [12], [28], [72]. In all selection rounds, 0.03% DDM was used as detergent instead of the commonly used Tween-20 in the standard ribosome display buffer WBT-BSA, containing 50 mM Tris-acetate pH 7.5, 150 mM NaCl, 50 mM MgOAc, and 0.5% BSA. For the DARPin selection against vanadate-trapped bLmrCD_AviC_, the protein was incubated with 1 mM ATP and 1 mM Na_3_VO_4_ (freshly boiled as 100 mM stock, pH 9–10) prior to (1 h on ice) and during the incubation with the ribosomal complexes. For the first three rounds, the selection was carried out using the surface panning method by immobilizing bLmrCD_AviC_ via neutravidin on a solid support as described in the protocol of Zahnd et al. [72]. The washing times before mRNA elution, were set to 5, 2×15 and 2×30 min in the first, the second and the third selection round, respectively. The fourth selection round was carried out with the solution panning method [31]. 60 nM of bLmrCD_AviC_ was added to the stabilized DARPin *in vitro* translation mixture (260 µl) and panned for 90 min. Streptavidin-coated magnetic beads (20 µl suspension Dynabeads MyOne Streptavidin T1, Invitrogen) were used to capture the biotinylated bLmrCD_AviC_ with bound ribosomal complexes during 15 min. The beads were rinsed twice with 300 µl WBT-BSA containing 0.03% β-DDM (WBT-BSA-DDM), placed into a fresh tube, and washed for 30 min. After another tube change and another 30 min of washing, the mRNA was eluted and purified according to the standard protocol [72].

### Crude Cell Extracts and ELISA

The pools of DARPins from the 4^th^ selection round were expressed from the vector pQE30_myc5_ [31] in *E. coli* XL-1 Blue yielding DARPins carrying an N-terminal RGS-His6 tag (with the protein sequence MRGSHHHHHH) and a C-terminal Myc5-tag (with five times the sequence MEQKLISEEDLNE). DARPin-containing crude cell extracts were used to identify LmrCD-specific binders by ELISA as described [31]. The DNA sequences of all identified DARPins have been deposited in GenBank under the accession numbers JQ425604-JQ425611.

### SEC of Isolated DARPins and the LmrCD-DARPin Complexes

The Myc5-tag fusion with the DARPins leads to the formation of higher oligomeric species (not shown), and the DARPins were therefore sub-cloned into the pQE30 vector (Qiagen) devoid of a Myc-tag for further analysis and purified via Ni^2+^-NTA chromatography and SEC (Superdex 200 10/300 GL, GE Healthcare) according to standard procedures [14]. For the quantification of the stoichiometric compositions of the LmrCD-DARPin complexes, Ni^2+^-NTA purified LmrCD (10 µM) was mixed with a twofold excess of freshly gel-filtrated DARPin and incubated for 30 min. The protein mixture was separated by SEC (Superdex 200 PC3.2/30, GE Healthcare), after which fractions were analyzed by on-chip protein analysis according to the manufacturer’s protocol (Protein 80 Kit, Agilent Technologies).

### Surface Plasmon Resonance

The affinities of selected DARPins towards detergent purified bLmrCD_AviC_ were determined by surface plasmon resonance on a Biacore T100 machine (GE Healthcare). Because initial SRP measurements in a buffer containing 0.03% DDM were difficult to interpret, the dissociation constants were determined in the presence of Tween-20 instead. To test the stability of LmrCD in Tween-20, DDM was replaced with highly pure Tween-20 (Anapoe-20, 0.05%, Anatrace) in the washing and elution step during LmrCD purification by Ni^2+^-NTA chromatography. LmrCD purified using Tween-20 exhibited an ATPase activity of 297±24 nmol/min/mg of protein and its SEC elution profile was indistinguishable from the one obtained with DDM (not shown). For the SRP measurement, the detergent was changed from DDM to Tween-20 after the immobilization of bLmrCD_AviC_ on the Biacore chip, which lead to highly accurate and undisturbed measurements. The target protein was purified freshly as described above and 600 response units (RU) were immobilized in flow cell 2 of a streptavidin-coated SA chip (GE Healthcare), whereas flow cell 1 was used for referencing. Affinities were determined in 20 mM Tris/HCl pH 7.5, 150 mM NaCl containing 0.05% (v/v) Tween-20 at 10°C and a flow rate of 20 µl/min. The DARPin concentration was determined by OD_280_ using a NanoDrop1000 Photospectrometer and calculated based on theoretical extinction coefficients (www.expasy.ch/tools/protparam.html). For each DARPin, a 3-fold dilution series of six different concentrations were used for the kinetic measurements (concentration ranges: 0.1 nM–72.9 nM for α-LmrCD#1, α-LmrCD#2; 0.3 nM –218.7 nM for α-LmrCD#3, α-LmrCD#5 and DARPin_Act3; 1 nM –729 nM for α-LmrCD#4; 3 nM - 2187 nM for DARPin_Act2). Every DARPin concentration was injected twice starting with the lowest concentration, increasing to the maximal concentration and then decreasing back to the lowest concentration. The association and dissociation phases were set as follows (the first number denotes association time/the second number denotes dissociation time): α-LmrCD#1, α-LmrCD#2 and DARPin_Act2 (700 s/2400 s); α-LmrCD#3 (240 s/1000 s); α-LmrCD#4, α-LmrCD#5 and DARPin_Act3 (400 s/1200 s). The data were best fitted using a two-state reaction model. This model assumes that the DARPin (A) and LmrCD (B) form an initial complex (AB) with an association rate constant k_a1_ (in M^−1^ s^−1^) and a dissociation rate constant k_d1_ (in s^−1^). This initial complex (AB) is then converted into an alternative complex (AB*) with the association rate constant k_a2_ (in s^−1^) and a dissociation rate constant k_d2_ (in s^−1^). In this model, the dissociation constant *K*_D_ (M) is calculated using the following equation:
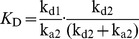
However, a control experiment in which a saturating concentration of a DARPin was injected for varying times revealed, that the two-state reaction model is inappropriate (see Results and Figure S3). Therefore, the data were fitted using a simple 1∶1 binding model and the dissociation constant *K*_D_ was calculated using the following equation in which k_a_ is the association rate constant and k_d_ the dissociation rate constant:



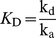
In addition, the steady-state response units at the end of each injection (i.e. when association and dissociation are in equilibrium) were plotted against the injected DARPin concentration (Figure 7D). The equilibrium constant *K*_D,eq._ was determined by non-linear regression using an equilibrium binding equation equivalent to the Michaelis-Menten equation in which R denotes the SPR response at equilibrium, R_max_ denotes the maximal SPR response and [DARPin] is the DARPin concentration:



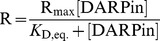



### Functional Screening in *L. lactis*

The control DARPin E3_5 [14] was cloned into the lactococcal vector pNZ8048 from which it was expressed in *L. lactis*. During the course of the study, DNA sequencing of the pNZ8048 clone of the control DARPin E3_5 revealed the replacement of the second repeat with the duplicated sequence of the third repeat in a recombination event. This variant of E3_5 (E3_5*) was monomeric (not shown) and was used as control DARPin in the functional experiments in *L. lactis*. For the functional screening of the DARPins in *L. lactis*, the DARPin pools of the 4^th^ selection round were expressed from pNZ8048 in the presence of nisin A (10 ng/ml) and daunomycin (10 µM or 18 µM to screen for inhibitors or activators, respectively). The plasmids encoding for potential inhibitory or activating DARPins were isolated, sequenced and retransformed into wildtype *L.* *lactis* NZ9000 and *L.* *lactis* NZ9000 Δ*lmrA* Δ*lmrCD.* Resistance towards daunomycin and Hoechst 33342 was determined by growing the cells at various drug concentrations. A pre-culture devoid of nisin (150 µl) was inoculated 1∶100 with an overnight culture, after which cells were grown for 210 min. The preculture was then diluted 1∶100 into medium containing 10 ng/ml nisin after which daunomycin was added to various concentrations, and growth of cells was allowed for 15 to 18 h. Final OD_660_ were measured and normalized by setting the final OD_660_ reached in the absence of drug to 100. Normalized values were plotted versus the daunomycin concentration. The curves were fitted with a 4-parameter sigmoidal equation in which *y* stands for the normalized final OD_660_, *y*_0_ describes the background OD_660_, *x* stands for the daunomycin concentration, *x*_0_ is the inflection point of the curve, and *a* and *b* are fitting parameters (SigmaPlot 10, default settings).
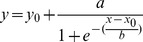



IC_50_ for daunomycin was defined as the daunomycin concentration at which the OD_660_ after growth for 15–18 h is half as high as in the absence of the drug.

### Transport Assay with BCECF-AM

*L. lactis* NZ9000 and *L. lactis* NZ9000 Δ*lmrA* Δ*lmrCD* harboring the expression plasmids for DARPin_Act2 and the unselected DARPin E3_5* were grown to an OD_660_ of 0.6 and induced for 2 h with 5 ng/ml nisin A. Cells were harvested and washed twice with ice-cold fluorescence buffer (50 mM potassium phosphate pH 7.0, 5 mM MgSO_4_). For the fluorescence measurements, the OD_660_ was adjusted to 0.5 and the cells were pre-energized by the addition of 0.5% glucose whilst stirring. Nigericin and valinomycin (1 µM each) were added prior to the addition of the fluorescent substrate. Non-fluorescent BCECF-AM was added at a final concentration of 0.2 µM. Subsequently, the formation of the fluorescent BCECF was monitored at excitation and emission wavelengths of 502 nm and 525 nm, respectively using slit widths of 2.5 nm and 4 nm, respectively.

### Quantification of LmrCD Production Levels in *L. lactis* by the Introduction of a V5-tag

The sequence of the V5 tag (with the protein sequence GKPIPNPLLGLDST) was introduced in frame with the genomic *lmrD* gene at its 3′-end in *L. lactis* using the Campbell-type recombination method [73]. The DNA sequence of the V5 tag containing the appropriate sticky overhangs was generated by annealing the oligonucleotides V5-tag_for and V5-tag_rev and cloned as double-stranded DNA fragment into pGEM_Avi cut with BamHI/NheI yielding pGEM_V5 and thereby replacing the Avi-tag sequence. An 860 bp stretch of chromosomal DNA downstream to the *lmrD* gene was amplified with the primers lmrD_V5_for1 and lmrD_V5_rev1 and introduced into pGEM_V5 using the restriction sites BamHI/XbaI resulting in pGEMLmrCD_V5_*. The last 1583 bp of *lmrD* were amplified with the primers lmrD_V5_for2 and lmrD_AviC_rev, cut with NcoI/XbaI and cloned in frame with the V5 tag sequence into pGEMLmrCD_V5_* cut with NcoI/NheI yielding pGEMLmrD_V5_. The DNA fragment on pGEMLmrD_V5_ containing the V5 tag sequence flanked by a part of *lmrD* and a stretch of DNA downstream of the *lmrD* gene on the *L. lactis* chromosome was sub-cloned into pORI280 via NcoI/XbaI and transformed into *E. coli* EC1000 (repA^+^) resulting in the plasmid pORI280LmrD_V5_ [73], [74]. Wildtype *L. lactis* NZ9000 was transformed with pORI280LmrD_V5_ as described [55] yielding three blue colonies after 3 days of incubation at RT. PCR analysis of the chromosomal DNA revealed that two of these clones were the result of the Campbell-type integration of pORI280LmrD_V5_. The second recombination step was performed by growing a positive clone for a total of 50 cell divisions in the absence of erythromycin and the subsequent screening for white colonies on M17 agar plates. Two white colonies were found (out of around 4000 colonies screened) and confirmed to encode the *lmrD* gene fused with the V5 tag sequence by Western blotting. This new strain was named *L. lactis* NZ9000 *lmrD_V5_.* The plasmids encoding the activator DARPins and the control DARPin E3_5* were transformed into *L. lactis* NZ9000 *lmrD_V5_*. A 1∶100 inoculated preculture of transformed cells was grown for 210 min in M17, 0.5% maltose, 5 µg/ml chloramphenicol and 50 µl thereof were used to inoculate 5 ml of the same medium containing 10 ng/ml nisin with or without daunomycin addition (14 µM for DARPin_Act3 and E3_5* and 28 µM for DARPin_Act1 and DARPin_Act2, respectively). Each sample was prepared in triplicates. The cultures were grown for 15 h and harvested by centrifugation. Cells were resuspended in 350 µl of 50 mM Na-HEPES (pH 7), 1 mM MgSO_4_, 10% (wt/v) glycerol, 1 mM PMSF, 25 µg/ml DNaseI and trace amounts of lysozyme. After the addition of glass beads (300 mg, 0.1-mm diameter), samples were disrupted in a FastPrep device (MP Fastprep-24, MB Biomedicals) twice for 30 s at force 6.5. Cell membranes were harvested by centrifugation (55000 g) resuspended by SDS-PAGE loading dye and the proteins were separated on a 10% tricine gel [75]. Each sample was analyzed on two SDS-PAGE gels, one dedicated to Western blotting and the other to the analysis of the protein amounts with SYPRO ruby staining (a total of 6 gels due to the triplicates). For the Western blotting analysis, the gels were blotted onto a nitrocellulose membrane (wet blotting) and blocked in TBST (TBS containing 0.1% Tween-20) supplied with 5% milk powder overnight. The anti-V5 antibody (Sigma, clone V5-10, 1∶3000 diluted in TBST) was panned for 160 min and the membrane was washed three times for 10 min with TBST. After incubation with a secondary anti-mouse HRP antibody (Jackson ImmunoResearch Laboratories, 1∶2500 in TBST) and another three washing steps, the Western blot signal was detected with a LAS-3000 imaging system (Fujifilm) using ECL reagent (PIERCE). The second SDS-PAGE gel corresponding to the samples analyzed by Western blot was stained with SYPRO ruby staining (Invitrogen) and the fluorescent signal was read with the LAS-3000 imaging system. The Western blots and the ruby-stained gels were quantified using the Aida software (Raytest). The data were normalized by setting the LmrD_V5_ level determined in cells expressing the control DARPin in the absence of drugs to 1. The standard deviations of the triplicates were calculated.

### DARPin Binding to Membrane Inserted Target Protein in ISOVs

For the production of membrane vesicles, LmrCD_AviC_ and AcrB_AviC_ were overproduced in *E. coli* C43 (DE3). Inside-out membrane vesicles (ISOVs) were obtained after cell disruption at 20000 psi (Constant Systems). ISOVs containing overexpressed LmrCD-GFP were prepared to determine the membrane vesicle orientation by cleaving off the GFP at the external side using 3C protease, followed by SDS-PAGE and quantification of the cleavage reaction using in-gel fluorescence of remaining LmrCD-GFP and cleaved GFP. Based on these experiments, ISOV preparations contained 10% or less membrane vesicles of the opposite (right-side-out) orientation. The membrane vesicles were diluted at a protein concentration of 0.2 mg/ml in 1 ml of TBS, pH 7.4. In a first set of experiments (Figure 8A) DARPins (350 nM) were allowed to bind for 40 min to the ISOVs. In a second set of experiments (Figure 8B), the DARPin_Act2 and α-LmrCD#3 used at concentrations of 0.35 µM, 1 µM and 2 µM were allowed to bind for 200 min. The membranes were harvested by centrifugation for 20 min at 55000 g. The pellets were resuspended with 800 µl of TBS to wash off unbound DARPins, spun again, after which the pellets were resuspended with SDS-PAGE loading dye (40 µl). Total membrane proteins in membrane vesicles, and bound DARPins, were separated by SDS-PAGE using tricine gels [75] and blotted onto nitrocellulose membranes. The protein mixture was separated by SDS-PAGE [75] and the bound DARPins were quantified by Western blotting using RGS-His antibody (Qiagen) and detection by ECL (PIERCE).

### Reconstitution of LmrCD and ATPase Activity Assay

Ni^2+^-NTA-purified LmrCD expressed in *L. lactis* was reconstituted at a protein:lipid ratio of 1∶50 (w/w) into acetone-washed and ether-extracted total *E. coli* lipids mixed with egg phosphatidylcholine (Avanti) in a ratio 3∶1 (w/w) in 50 mM K-HEPES pH 7.0 following published protocols [41], [76]. Where indicated, SEC-purified DARPins (2.5 µM) were added to the proteoliposomes and incubated in 50 mM K-HEPES pH 7 for 12 h. Daunomycin (where indicated) and MgSO_4_ (10 mM) were added shortly prior to the assay start. The ATPase assay was performed in 96-well PCR plates on the heating block of a PCR machine. 40 µl of reconstituted LmrCD (70 nM, including DARPins and daunomycin where appropriate) was added to 10 µl of 5-fold stock of highly pure ATP solution (SigmaUltra, 1 mM final concentration if not stated otherwise, dissolved in ddH_2_O adjusted to pH 7 using KOH) whilst the temperature was set to 4°C. The ATP hydrolysis reaction was initiated by changing the temperature to 30°C for 20 min and stopped by denaturing the samples at 80°C for 30 s. LmrCD mutated at the Walker B glutamate of the consensus composite ATPase site (LmrD_E587Q) was reconstituted and used for background subtractions. This mutation was shown previously and confirmed by us to be incapable of hydrolyzing ATP [47]. The amount of generated Pi was quantified colorimetrically using the malachite green/molybdate method [55]. The datapoints of the ATPase activities measured at increasing ATP concentrations (Figure 9C) were fitted with the 3 parameter Hill equation (Sigmaplot 10, default settings), in which *y* denotes the ATPase activity, *x* stands for the concentration of ATP, *a* corresponds to V_max_, *b* denotes the Hill coefficient, and *c* corresponds to *K*_m,app_.
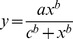



### Data Analysis

Statistical analyses were performed with the Student’s t-test with a 95% confidence interval for the sample mean. If not stated otherwise, error bars represent the standard deviation (SD).

## Supporting Information

Figure S1 **Preparation of biotinylated target proteins for the DARPin selections and ELISAs, and characterization of selected DARPins by SEC.** (A) SDS-PAGE analysis of purified LmrCD_AviC_. The protein bands corresponding to overproduced LmrCD_AviC_ are apparent in the total detergent-solubilized membrane fraction (lane 1). Pure protein is eluted from the Ni^2+^-NTA column (lane 2). (B) Ni^2+^-NTA purified LmrCD_AviC_ shown in (A) was *in vitro* biotinylated and separated by SEC to remove aggregated protein and excess biotin. Fractions of the peak at 12.50 ml corresponding to heterodimeric bLmrCD_AviC_ were used for the DARPin selections and ELISA (red bar). The strong peak at the void volume of the column (9 ml) besides aggregated LmrCD also contained genomic DNA that escaped from DNaseI treatment (as evidenced by the strong A_254_ signal relative to the A_280_ signal). (C, D), Gel filtration profiles of studied DARPins on Superdex 200 column. The maxima of the main peaks were as follows: (C) α-LmrCD#1∶16.84 ml; α-LmrCD#2∶15.11 ml; α-LmrCD#3∶16.37 ml; LmrCD#4∶16.80 ml; α-LmrCD#5∶17.01 ml; E3_5∶16.89 ml (D) DARPin_Act1∶10.32 ml; DARPin_Act2∶13.25 ml; DARPin_Act3∶16.38 ml. (E, F), SEC profiles of LmrC-GFP and LmrD-GFP (E) as well as of LmrD-NBD_AviN_ (F). The fractions indicated by the red bar were used for the ELISA shown in Figure 3B.
(TIF)

Figure S2 **Sequence alignment of the LmrCD-specific DARPins identified in this study.** The sequence of the consensus designed DARPin framework is given in the top line, where “x” stands for all amino acids except proline, glycine and cysteine and “y” stands for histidine, glutamine or tyrosine.
(TIF)

Figure S3 **SPR control experiment disfavors a two-state reaction model of DARPin binding to LmrCD.** The fits of the SPR sensograms were found to match better using a two-state reaction model instead of a 1∶1 binding model (see Materials and Methods). To test whether the two-state reaction model was appropriate for fitting, a saturating concentration of α-LmrCD#3 (400 nM) was injected onto a SPR SA-chip containing 600 RU of immobilized bLmrCD_AviC_ for 100 s, 200 s and 400 s (each injection was performed twice). The traces were superimposed at the starting point of the dissociation curve. DARPin dissociation is virtually identical irrespective of the duration of association time, indicating that the two-state reaction model is not appropriate. Therefore, all SPR data were fitted using a 1∶1 binding model (Figure 7C and Table 1).
(TIF)

Table S1 **Primers used in this study.**
(DOC)

Table S2 **Genetic constructs used in this study.**
(DOC)
