# Supplementary figures and images for: Tuning the Drug Efflux Activity of an ABC Transporter in vivo by in vitro Selected DARPin Binders
Source: PLoS One. 2012 Jun 4;7(6):e37845. doi: 10.1371/journal.pone.0037845 (PMC3366976; doi:10.1371/journal.pone.0037845)

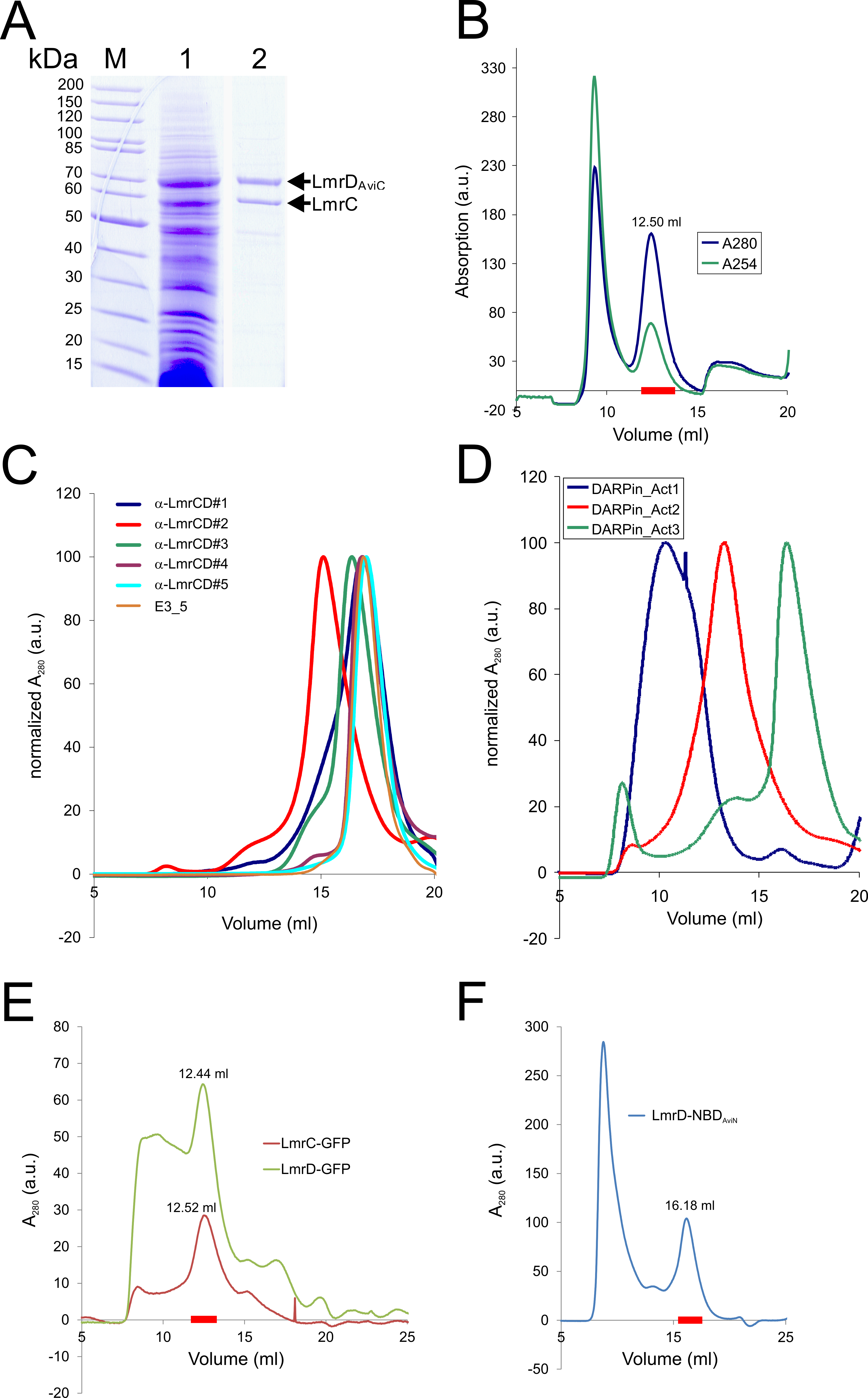

Supplement: Figure S1 — Preparation of biotinylated target proteins for the DARPin selections and ELISAs, and characterization of selected DARPins by SEC. (A) SDS-PAGE analysis of purified LmrCDAviC. The protein bands corresponding to overproduced LmrCDAviC are apparent in the total detergent-solubilized membrane fraction (lane 1). Pure protein is eluted from the Ni2+-NTA column (lane 2). (B) Ni2+-NTA purified LmrCDAviC shown in (A) was in vitro biotinylated and separated by SEC to remove aggregated protein and excess biotin. Fractions of the peak at 12.50 ml corresponding to heterodimeric bLmrCDAviC were used for the DARPin selections and ELISA (red bar). The strong peak at the void volume of the column (9 ml) besides aggregated LmrCD also contained genomic DNA that escaped from DNaseI treatment (as evidenced by the strong A254 signal relative to the A280 signal). (C, D), Gel filtration profiles of studied DARPins on Superdex 200 column. The maxima of the main peaks were as follows: (C) α-LmrCD#1∶16.84 ml; α-LmrCD#2∶15.11 ml; α-LmrCD#3∶16.37 ml; LmrCD#4∶16.80 ml; α-LmrCD#5∶17.01 ml; E3_5∶16.89 ml (D) DARPin_Act1∶10.32 ml; DARPin_Act2∶13.25 ml; DARPin_Act3∶16.38 ml. (E, F), SEC profiles of LmrC-GFP and LmrD-GFP (E) as well as of LmrD-NBDAviN (F). The fractions indicated by the red bar were used for the ELISA shown in Figure 3B. (TIF) [file pone.0037845.s001.tif]

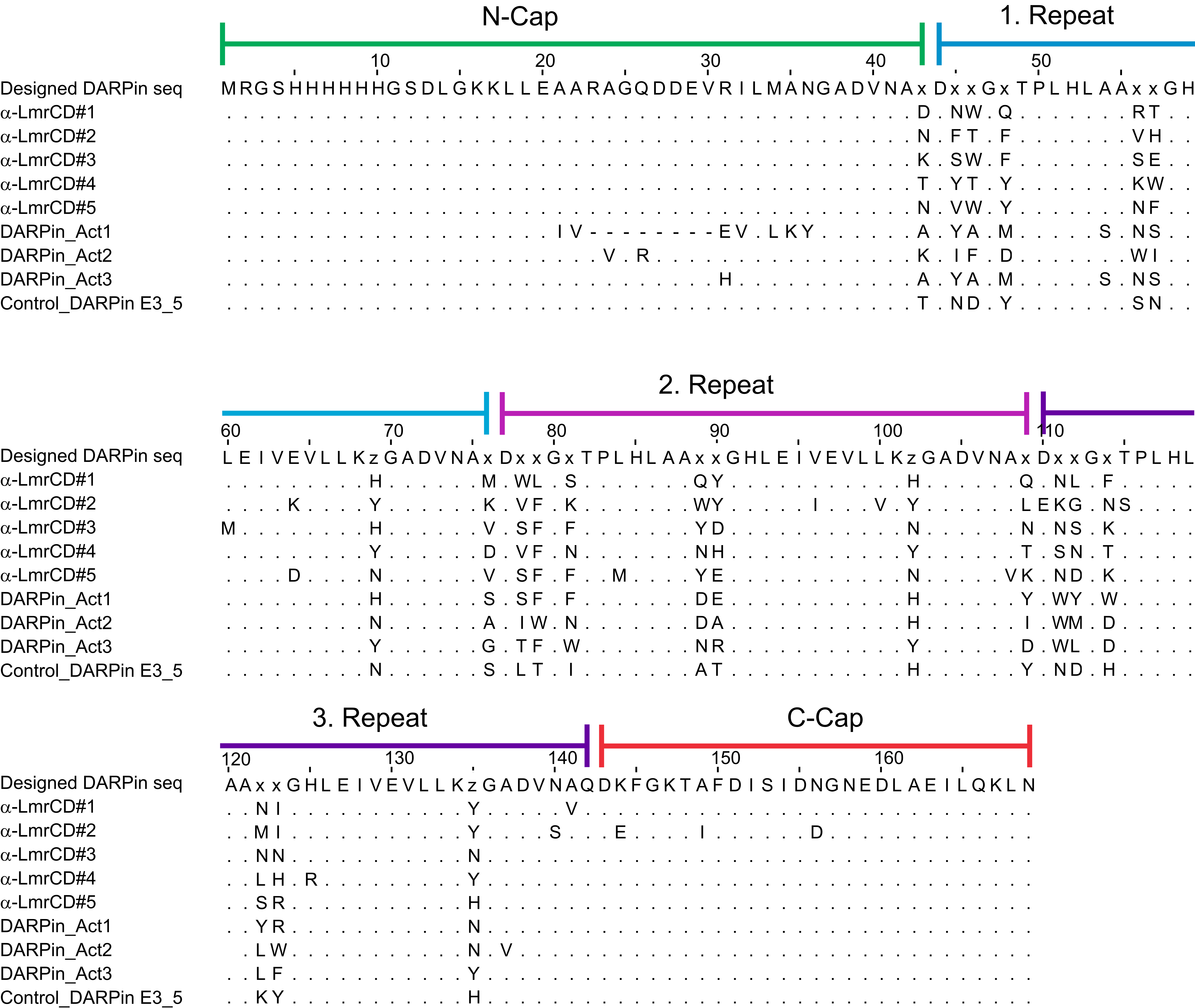

Supplement: Figure S2 — Sequence alignment of the LmrCD-specific DARPins identified in this study. The sequence of the consensus designed DARPin framework is given in the top line, where “x” stands for all amino acids except proline, glycine and cysteine and “y” stands for histidine, glutamine or tyrosine. (TIF) [file pone.0037845.s002.tif]

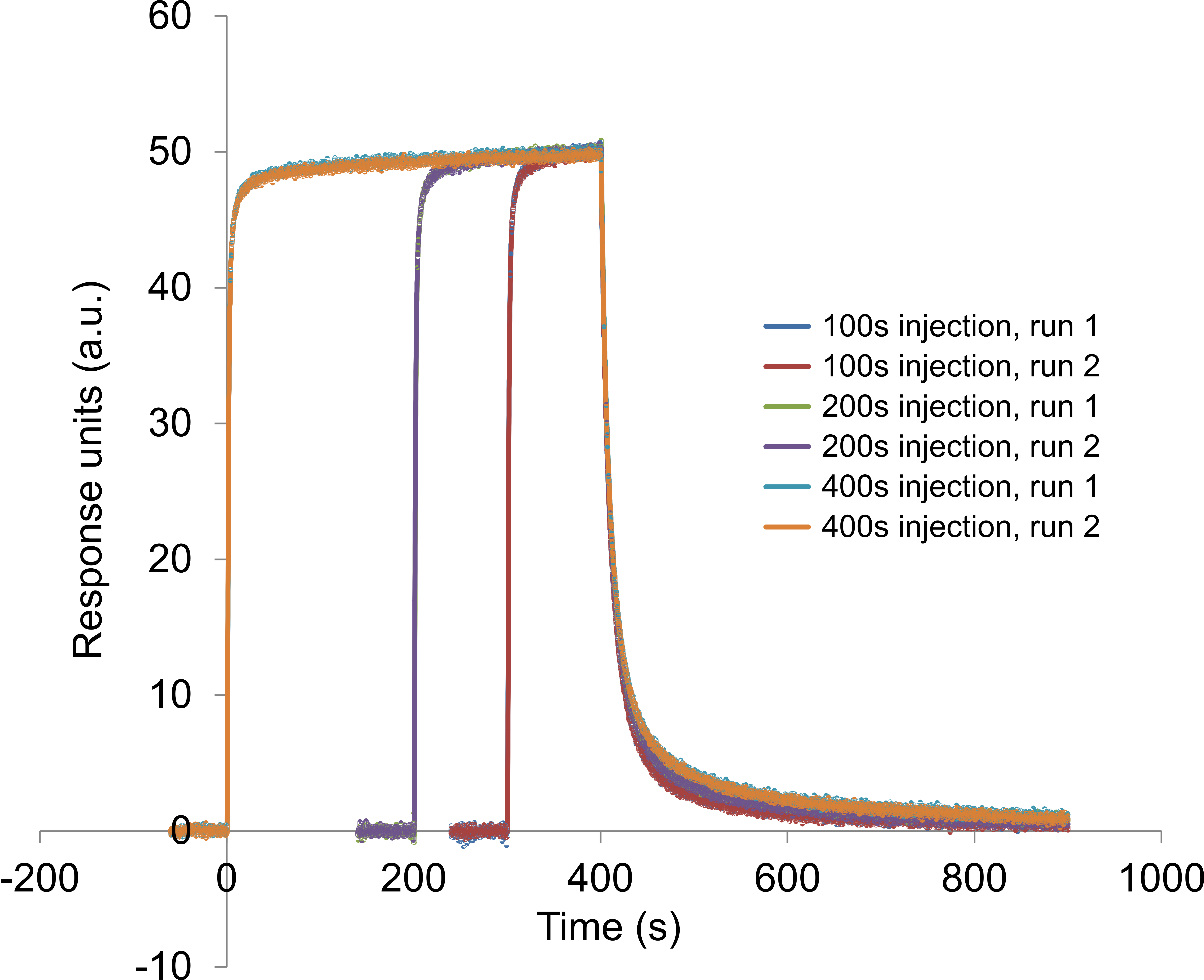

Supplement: Figure S3 — SPR control experiment disfavors a two-state reaction model of DARPin binding to LmrCD. The fits of the SPR sensograms were found to match better using a two-state reaction model instead of a 1∶1 binding model (see Materials and Methods). To test whether the two-state reaction model was appropriate for fitting, a saturating concentration of α-LmrCD#3 (400 nM) was injected onto a SPR SA-chip containing 600 RU of immobilized bLmrCDAviC for 100 s, 200 s and 400 s (each injection was performed twice). The traces were superimposed at the starting point of the dissociation curve. DARPin dissociation is virtually identical irrespective of the duration of association time, indicating that the two-state reaction model is not appropriate. Therefore, all SPR data were fitted using a 1∶1 binding model (Figure 7C and Table 1). (TIF) [file pone.0037845.s003.tif]
