# Supplementary material for: Tuning the Drug Efflux Activity of an ABC Transporter in vivo by in vitro Selected DARPin Binders
Source: PLoS One. 2012 Jun 4;7(6):e37845. doi: 10.1371/journal.pone.0037845 (PMC3366976; doi:10.1371/journal.pone.0037845)
Supplement: Table S1 — Primers used in this study. (DOC) [file pone.0037845.s004.doc]

**Table S1** Primers used in this study

| Primer | Sequence |
| --- | --- |
| avitag_for | 5’-CAT GGG CGC TAG CGG TCT GAA CGA TAT CTT CGA AGC TCA GAA AAT CGA ATG GCA CGA AGG ATC CTA AT-3’ |
| avitag_rev | 5’- CTA GAT TAG GAT CCT TCG TGC CAT TCG ATT TTC TGA GCT TCG AAG ATA TCG TTC AGA CCG CTA GCG CC -3’ |
| lmrCD_DecaHisN_AviC_for | 5’-ATC CAT GGG GCA TCA CCA TCA CCA TCA CCA TCA CCA TCA TAT GAT TTT CAA ATC AAT CAT GAA GCA TAA ATG-3’ |
| lmrCD_AviC_rev | 5’- TAT CTA GAT TCA AAA ACG AAT TGA TTA TGA TAA AG -3’ |
| lmrCD_DecaHisN_Presc_for | 5’-TAT ATC ATA TGC TAG AAG TTC TGT TCC AGG GGC CGG CAG GTG CTG GAG CAA TTT TCA AAT CAA TCA TGA AGC ATA AAT G-3’ |
| lmrCD_rev | 5’-TAT CTA GAT TAT CAT TCA AAA ACG AAT TGA TTA TGA TAA AG-3’ |
| msbA_DecaHisN_for | 5’-ATA TCC ATG G GC CAT CAC CAT CAC CAT CAT CAT CAT CAT CAC AGC-3’ |
| msbA _AviC_rev | 5’-ATA TGC TAG CTT GGC CAA ACT GCA TTT TGT G-3’ |
| acrB_HisC_AviC_for | 5’-ATC CAT GGG GCC TAA TTT CTT TAT CGA TCG CCC G-3’ |
| acrB_HisC_AviC_rev | 5’-ATG CTA GCG TGA TGG TGA TGG TGA TGG TGA TGC TGC AGA TGA TGA TCG ACA GTA TGG CTG TG-3’ |
| EWT5 | 5’-TTC CTC CAT GGG TAT GAG AGG ATC GCA TCA CCA TCA CCA TCA CGG ATC CGA CCT GGG-3’ |
| MTS46 | 5’-GCA TAA GCT TCA TTA GTT CAG TTT CTG CAG GAT TTC AGC CAG GTC-3’ |
| E3_5_NcoI_for | 5’-AAT CCA TGG GAA GAG GAT CGC ATC ACC ATC AC-3’ |
| V5-tag_for | 5’-CTA GCG GTA AGC CTA TCC CTA ACC CTC TTC TTG GTC TTG ATT CTA CAT AAG-3’ |
| V5-tag_rev | 5’-GAT CCT TAT GTA GAA TCA AGA CCA AGA AGA GGG TTA GGG ATA GGC TTA CCG-3’ |
| lmrD_V5_for1 | 5’-ATG GAT CCG AAA AAC TTG TTT GAA TAA AAG AAA TAA AAA CTA C-3’ |
| lmrD_V5_rev1 | 5’-TAT CTA GAT GAG CTC CGT GCA TTT CAT CTA TC-3’ |
| lmrD_V5_for2 | 5’-TAC CAT GGA GCA CTT CTA CAT TCA TGA AAG GAA TG-3’ |
