# Supplementary material for: Tuning the Drug Efflux Activity of an ABC Transporter in vivo by in vitro Selected DARPin Binders
Source: PLoS One. 2012 Jun 4;7(6):e37845. doi: 10.1371/journal.pone.0037845 (PMC3366976; doi:10.1371/journal.pone.0037845)
Supplement: Table S2 — Genetic constructs used in this study. (DOC) [file pone.0037845.s005.doc]

**Table S2** Genetic constructs used in this study

| Plasmid name | Explanation |
| --- | --- |
| pGEM_avi | pGEM vector containing the Avi-tag linker as basis for further cloning |
| pGEMLmrCD_AviC_ | *lmrCD* cloned into pGEM containing an N-terminal His_10_ tag and a C-terminal Avi-tag sequence |
| pBADLmrCD_AviC_ | *lmrCD* cloned into pBAD24 containing an N-terminal His_10_ tag and a C-terminal Avi-tag sequence |
| pNZLmrCD_AviC_ | *lmrCD* cloned into pNZ8048 containing an N-terminal His_10_ tag and a C-terminal Avi-tag sequence |
| pGEMLmrCD | *lmrCD* cloned with a N-terminal His_10_ tag and a prescission cleavage site into pGEM |
| pBADLmrCD | *lmrCD* cloned with a N-terminal His_10_ tag and a prescission cleavage site into pBAD24 |
| pNZLmrCD | *lmrCD* cloned with a N-terminal His_10_ tag and a prescission cleavage site into pNZ8048 |
| pGEMMsbA_AviC_ | *msbA* cloned into pGEM with a N-terminal His_10_ tag and a C-terminal Avi-tag sequence |
| pBADMsbA_AviC_ | *msbA* cloned into pBAD24 with a N-terminal His_10_ tag and a C-terminal Avi-tag sequence |
| pGEMAcrB_AviC_ | *acrB* cloned into pGEM with a N-terminal His_10_ tag and a C-terminal Avi-tag sequence |
| pBADAcrB_AviC_ | *acrB* cloned into pBAD24 with a N-terminal His_10_ tag and a C-terminal Avi-tag sequence |
| pNZE3_5 | control DARPin E3_5 cloned into pNZ8048 |
| pGEM_V5 | pGEM vector containing the V5 tag sequence for further cloning |
| pGEMLmrD_V5_* | pGEM vector containing the V5 tag sequence and 860 bp of chromosomal sequence 3’ to the *lmrD* gene |
| pGEMLmrD_V5_ | pGEMLmrD_V5_* containing the last 1583 bp of *lmrD* cloned in frame with the V5 tag sequence |
| pORILmrD_V5_ | pORI280 containing 1583 bp of *lmrD* cloned in frame with the V5 tag sequence followed by 860 bp of chromosomal sequence 3’ to the *lmrD* gene |
